# Atlas of Cancer Signalling Network: a systems biology resource for integrative analysis of cancer data with Google Maps

**DOI:** 10.1038/oncsis.2015.19

**Published:** 2015-07-20

**Authors:** I Kuperstein, E Bonnet, H-A Nguyen, D Cohen, E Viara, L Grieco, S Fourquet, L Calzone, C Russo, M Kondratova, M Dutreix, E Barillot, A Zinovyev

**Affiliations:** 1 Institut Curie, Paris, France; 2 INSERM, U900, Paris, France; 3 Mines ParisTech, Fontainebleau, France; 4 Sysra, Paris, France; 5 Ecole Normale Supérieure, IBENS, Paris, France; 6 CNRS, UMR8197, Paris, France; 7 INSERM, U1024, Paris, France; 8 CNRS, UMR3347, Orsay, France; 9 INSERM, U1021, Orsay, France

## Abstract

Cancerogenesis is driven by mutations leading to aberrant functioning of a complex network of molecular interactions and simultaneously affecting multiple cellular functions. Therefore, the successful application of bioinformatics and systems biology methods for analysis of high-throughput data in cancer research heavily depends on availability of global and detailed reconstructions of signalling networks amenable for computational analysis. We present here the Atlas of Cancer Signalling Network (ACSN), an interactive and comprehensive map of molecular mechanisms implicated in cancer. The resource includes tools for map navigation, visualization and analysis of molecular data in the context of signalling network maps. Constructing and updating ACSN involves careful manual curation of molecular biology literature and participation of experts in the corresponding fields. The cancer-oriented content of ACSN is completely original and covers major mechanisms involved in cancer progression, including DNA repair, cell survival, apoptosis, cell cycle, EMT and cell motility. Cell signalling mechanisms are depicted in detail, together creating a seamless ‘geographic-like' map of molecular interactions frequently deregulated in cancer. The map is browsable using NaviCell web interface using the Google Maps engine and semantic zooming principle. The associated web-blog provides a forum for commenting and curating the ACSN content. ACSN allows uploading heterogeneous omics data from users on top of the maps for visualization and performing functional analyses. We suggest several scenarios for ACSN application in cancer research, particularly for visualizing high-throughput data, starting from small interfering RNA-based screening results or mutation frequencies to innovative ways of exploring transcriptomes and phosphoproteomes. Integration and analysis of these data in the context of ACSN may help interpret their biological significance and formulate mechanistic hypotheses. ACSN may also support patient stratification, prediction of treatment response and resistance to cancer drugs, as well as design of novel treatment strategies.

## Introduction

Understanding of tumorigenesis has drastically improved recently owing to extensive studies of the implicated molecular mechanisms.^1^ This knowledge mostly exists in a human-readable form, fragmented in scientific publications. This circumstance hampers the delineation of a global picture of cancer biology and prevents from efficient integration of high-throughput data and application of bioinformatics and systems biology approaches.^2^ The effort towards systematic collection and formalization of the knowledge about molecular interactions in a computer-readable form gave birth to pathway databases.^3^ Nevertheless, there is no up-to-date pathway database that systematically depicts molecular processes specifically implicated in cancer.

Most existing pathway databases provide a view on molecular mechanisms as disconnected processes, splitting their content into ‘canonical pathway' representations. However, cancerogenesis affects simultaneously multiple cellular processes and their crosstalk. Therefore, cancer research can benefit from the reconstruction of cellular signalling in the form of a comprehensive map, representing the complexity of pathway crosstalk^4^ manifested by co-participation, interaction or co-regulation of molecular entities in several cell signalling processes. Understanding connections between molecular mechanisms is important for determining potential therapeutic intervention points.^5, 6^

To enable reconstruction of comprehensive cell signalling maps, the common standard for depicting biological knowledge, the Systems Biology Graphical Notation has been developed.^7^ In addition, handful software tools for charting the large biological maps, such as CellDesigner,^8^ CellIllustrator^9^ and others, stimulated the community to create large comprehensive maps of molecular interactions implicated in various biological processes. Therefore, large maps of signalling pathways,^10, 11, 12, 13, 14, 15, 16^ of complex metabolic processes,^17^ as well as maps specific for certain cell types^18, 19^ or non-mammalian organisms^19, 20, 21^ have appeared. Comprehensive maps representing biological mechanisms that are implicated in particular diseases such as Alzheimer disease or rheumatoid arthritis have also been charted recently.^22, 23^

Several attempts have been undertaken to connect cancer-driver genes into relatively large networks or to represent them in pathway databases.^24, 25^ Cancer Cell Map database was created and included into the Pathway Commons network biology resource.^26^ However, those resources are far from being comprehensive representations of cancer-related processes. Therefore, there is the need for a global and detailed representation of molecular mechanisms involved in cancer, depicting the latest discoveries in this rapidly evolving field.

We have thus constructed the Atlas of Cancer Signalling Network (ACSN), which describes in detail signalling and regulatory molecular processes that occur in a healthy mammalian cell but that are frequently deregulated during cancerogenesis. ACSN is characterized by several features whose combination makes it unique: (i) it is a comprehensive description of cancer-related mechanisms retrieved from the most recent literature; (ii) its content is represented as an interactive global map that can be browsed with Google Maps interface; (iii) it has an online discussion forum, allowing users to provide feedback; and (iv) it has a built-in tool box for visualization and analysis of high-throughput data in the context of the comprehensive signalling network.

## Results

### ACSN concept and features

#### ACSN is an interconnected modular ‘geographical-like' global map of cancer signalling

**Figure 1:**
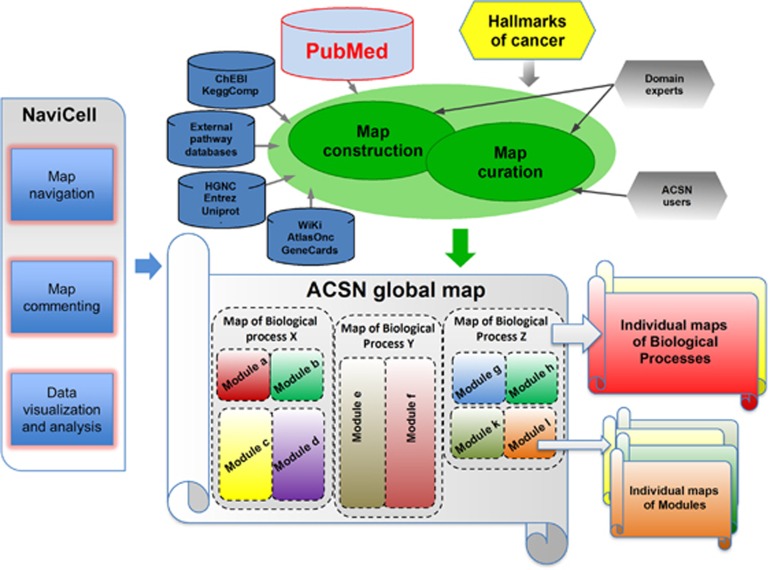
Structure and content of ACSN resource. The scheme demonstrates the concept of ACSN construction starting from the cancer hallmarks: collecting information about molecular mechanisms underlying those hallmarks from scientific publications and manually depicting them in the global map of ACSN and further supporting by consulting the information from the external pathway databases. ACSN is hierarchically organized into three levels: the seamless global map divided into the interconnected biological process maps that are further decomposed into interconnected module maps. ACSN can be exploited through web-based NaviCell interface allowing map navigation using Google Maps engine, map commenting via associated blog system and user omics data visualization and analysis.

**Figure 2:**
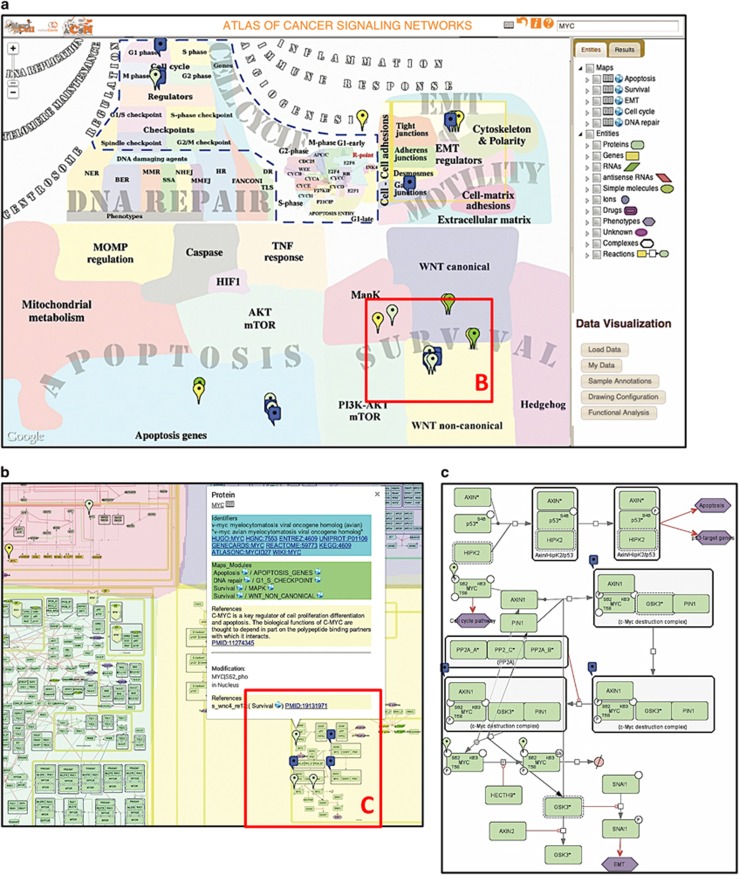
ACSN browsing and data analysis features. ACSN interface. (**a**) The NaviCell-powered Google Maps-based ACSN interface includes map window, selection panel, data analysis panel and upper panel. Such elements as zoom bar, markers and callout window (in panel **b**) are part of the Google Maps engine. Querying ACSN is possible via search window or by checking on the entity in the list of entities in the selection panel that will drop markers all over the map (for example, MYC). (**b**) Zoom in at a fragment of ACSN global map. Markers are preserved through all zoom levels. Clicking on a marker opens a callout window containing three sections: ‘Identifiers' with links to external databases; ‘Maps_Modules' with links to ACSN maps and modules where the entity (for example, MYC) is found, ‘References' with links to PubMed, and comments from the map managers. Clicking on the ‘globe' icon opens the corresponding map, clicking on the ‘book' icon opens the blog with corresponding post with detailed information about the entity. (**c**) Zoom in at a fragment of a module. The zoom of the WNT-non-canonical module, part of the survival map, shows the most detailed level of the molecular mechanism representation.

ACSN graphically depicts molecular mechanisms that occur in normal, healthy mammalian cells but that are frequently modified in cancer, underlying the hallmarks of cancer.^1^ Currently, ACSN covers signalling pathways involved in DNA repair, cell cycle, cell survival, cell death, epithelial-to-mesenchymal transition (EMT) and cell motility. The information included in ACSN is organized in three hierarchical levels: (i) the seamless global map with all molecular interactions, (ii) containing five maps of biological processes, and (iii) in turn comprising 52 functional modules (Figure 1). By analogy with geographical maps, ACSN can be viewed as a global map of cancer signalling divided into ‘continents' (that is, maps), each representing a particular biological process. Each continent is further divided to ‘countries' (that is, modules). Such structure allows visualization and exploration of either the whole network or its constitutive subparts (Figure 2). ACSN represents a large biochemical reaction network of 4826 reactions covering 2371 proteins, 5975 chemical species and supported by information from 2919 articles (Table 1).

#### ACSN is manually constructed using standard graphical notation

ACSN is based on the information about molecular mechanisms manually extracted from scientific literature and external databases by map managers. The maps are constructed using Systems Biology Graphical Notation language^7^ as it is implemented in CellDesigner tool^8^ that ensures compatibility of ACSN maps with various tools for network analysis, data integration and network modeling.

Hierarchical organization of ACSN (Figure 1) reflects the principle of literature curation for its construction. The major review papers in the field were the basis for defining the borders of the ACSN maps. The key canonical pathways, as they are depicted in the major pathway databases, represent the basis for depicting modules in ACSN, reflecting the consensus view of the scientific community on the pathways. The most detailed level of ACSN depicts information extracted from the recent literature with the requirement that the interactions and processes included to this level of ACSN are supported by several (at least two) independent investigations. The process of construction and curation of ACSN maps, including graphical standard, data model, rules of literature selection, data input from other databases and assignment of confidence scores, are detailed in the Materials and methods section.

Each map in ACSN covers hundreds of molecular players, biochemical reactions and causal relationships between the molecular players and cellular phenotypes. Each molecular player and each reaction on the map is annotated with the PubMed references and cross-referenced with other molecular biology databases. The notes of map managers can be added (Figure 2, Supplementary Figures S2 and S3) in a free format. After the construction, the maps are critically reviewed by the experts in the corresponding fields. Exchange of information with experts is facilitated by an associated web-blog, where comments regarding each component of the map can be posted (Supplementary Figures S2 and S3).

### ACSN content and representation of cancer-related molecular mechanisms

The biological mechanisms represented in ACSN are briefly described below and their correspondence to the hallmarks of cancer is made explicit. A more detailed description can be found at ACSN website (http://acsn.curie.fr).

#### Cell cycle

One of the main traits of cancer is uncontrolled cells' proliferation, caused by deregulation of the cell cycle machinery. The ‘Cell cycle' map of ACSN is connected to ‘Sustaining proliferative signalling' and ‘Evading growth suppressors' cancer hallmarks mainly through cyclin D-mediated signalling. The Cell cycle map, previously published in Calzone *et al.*^10^ is composed of 17 interconnected functional modules drawn as a circle where the main players are organized in a temporal manner demonstrating propagation of cell cycle through four phases. Transcriptional targets of E2F transcription factors are depicted above the circle.

#### DNA repair

DNA repair mechanisms and their coordination with cell cycle through checkpoints are frequently deregulated in familial and sporadic cancers.^27, 28^ This might lead to the appearance of genomic instability and justifies treatment by conventional DNA damaging chemotherapy drugs. Deregulation of DNA repair underlies ‘Genome instability and mutation' cancer hallmark. In ACSN, the knowledge of DNA repair machinery and its connection to the cell cycle checkpoints is represented as layers. The upper layer depicts cell cycle, the middle layer represents cell cycle checkpoints coordinating the crosstalk between the cell cycle and the DNA repair machinery represented in the lower layer. The DNA repair layer is organized in 10 individual (though interconnected) DNA repair pathways.

#### Cell survival

Signalling mechanisms involved in cell survival are widely exploited by cancer, ensuring the viability of a cell in diverse conditions, such as hypoxia, autophagy, anoikis and lack of nutrients.^29, 30, 31^ Cell survival together with apoptosis (see below) corresponds to the ‘Resisting cell death' hallmark of cancer. Cell survival mechanisms determine ‘Sustaining proliferative signalling' and 'Evading growth suppressors' cancer hallmarks through multiple signalling cascades upstream of the cell cycle. In ACSN, representation of cell survival mechanisms is divided into four major modules. Mechanisms depicted on the survival map are involved in modulation of the antiapoptotic, proliferation, EMT and migration processes. Cell survival description in ACSN is visually organized as a chessboard with squares that makes the connection between individual module maps more straightforward.

#### Apoptosis

Programmed cell death is one of the fundamental cellular processes that has a crucial role in tumorigenesis. Frequent deregulation in programmed cell death machinery, especially apoptosis, is one of the central hallmarks of cancer, ‘Resisting cell death'.^32, 33^ ACSN contains seven modules devoted to mechanisms of apoptosis as intrinsic and extrinsic regulatory mechanisms and to the core of the apoptotic machinery. The interplay between apoptosis and part of glucose metabolism (Warburg effect) is represented,^34^ which creates a link to another cancer hallmark, ‘Deregulating cellular energetics'. In ACSN, apoptosis description is visually organized in several cell compartments with the structure of mitochondrial membranes represented in more detail.

#### EMT and cell motility

EMT and cell motility is a normal process during embryonic development^35^ that can be activated in wound healing and fibrosis. In cancer, EMT promotes invasion of tumour cells into surrounding stromal tissues. Description of cell motility-related processes corresponds to ‘Activating invasion and metastasis' cancer hallmark. In ACSN, the EMT and cell motility map is composed of five interconnected functional modules with the core EMT regulators placed in the centre of the map. Graphically, the map represents a cell directly interacting with a neighbour cell and with extracellular matrix.

### ACSN web interface: access, navigation, visualization, and maintenance

The ACSN website provides extensive documentation on ACSN, including online tutorials, user guides and videos. The content of the atlas is available for downloading in several exchange formats (see Materials and methods). The user-friendly interface connects together the global Atlas map, the individual maps of biological processes and the functional module maps. The visualization and navigation of maps are supported by the NaviCell web-based environment^36^ empowered by Google Maps engine. Navigation features such as scrolling, zooming, markers, callout windows and zoom bar, are adopted from the Google Maps interface. All map components are ‘clickable', making the map interactive (Figure 2). ACSN allows querying for single or multiple molecules using the search window with an option of advanced search. Alternatively, the entity of interest can be found in the selection panel or by clicking directly on the map (Figure 2a).

ACSN is a large and detailed map of molecular interactions representing multiple distant crosstalks between molecular processes. This richness is a ‘double-edged sword', because the density of the presented information makes the navigation through the map difficult. This problem is addressed in ACSN in four complementary ways.

First, exploration of the content is facilitated by the hierarchical modular structure of ACSN. Shuttling between the global ACSN map to individual maps of biological processes and further to functional module maps is possible. It allows visualization of only a limited number of molecular species and reactions related to a particular molecular mechanism (Figures 1, 2b and c).

Second, the semantic zooming feature in ACSN simplifies navigation through the large maps of molecular interactions, showing readable amount of details at each zoom level. Gradual exclusion of details allows exploration of map content from the detailed towards the top-level view (Supplementary Figure S4). Pruning of maps helps to eliminate non-essential information, highlighting the core processes corresponding to the canonical pathways. The principle of pruning can be understood from the ACSN representation of the Cell cycle map or from the mitogen-activated protein kinase (MAPK) module of Cell survival maps (Supplementary Figure S5).

Third, to facilitate exploration of individual entities of the map, the function of selecting and highlighting only species of interest is available in the ACSN user interface (detailed in the user guide at the ACSN website). It is also possible to select and highlight neighbours of a molecular species of interest. This way, the connectivity of the selected entities through the map can be studied. Step-wise enlarging of the neighbourhood coverage can help to follow the propagation of signalling from one molecular entity to another (Supplementary Figure S6).

The fourth way to explore the map is via detailed annotation post of each component of the map that can be accessed through the callout window or from the selection panel (Figure 2 and Supplementary Figure S3). Annotations contain extended information on the entities, such as involvement in ACSN maps and modules; participation in complexes; participation in reactions and their regulation and so on. Each component listed in the annotation post is a hyperlink leading to the corresponding map entity. Shuttling between the annotation post and the map facilitates the exploration of ACSN. In addition, annotation posts contain links to directly access external resources. Finally, users can leave comments via an integrated blog system that helps to map maintenance (Supplementary Figures S2 and S3).

The field of oncology evolves rapidly, therefore ACSN maps require regular updates. The process of maintenance involves map managers that regularly collect the users' comments posted in the blog of ACSN, check the latest scientific literature and update the maps and the annotations accordingly. An automated procedure supports update of ACSN and archives older versions of posts including users' comments, thus providing traceability of all changes on the maps and all discussions in the blog (Supplementary Figures S2 and S3).

#### Comparison of ACSN to other pathway databases

Because of its focus on cancer-related processes, ACSN is smaller in absolute size compared with other general-scope pathway databases (Supplementary Table S1). ACSN does not cover as wide range of cell signalling and metabolic pathways as REACTOME^37^ or KEGG PATHWAY.^38^ However, one can suggest that focussing on fewer cellular processes allows performing a more detailed description of molecular mechanisms resulting in a denser and richer interaction network. We compared ACSN with two pathway databases that have similar structure and level of details: REACTOME^37^ and National Cancer Institute Pathway Interaction Database (NCI PID).^25^ BiNoM plugin of Cytoscape was used to process BioPAX 3.0 files,^39, 40^ storing the content of the three pathway databases compared. We only used protein-related parts of each database and excluded the metabolic reactions involving small molecules only. We have found that each pathway database among the three is characterized by a specific distinguishing feature. REACTOME is the largest among the three pathway databases in terms of the number of proteins and reaction. However, ACSN is almost three times denser in terms of the number of reactions per protein and is comparable to NCI PID in this aspect. NCI PID is the densest among the three databases in terms of the number of protein complex species described per protein (Supplementary Figure S7A).

We further extracted only the reaction graphs from the three databases and analysed their largest connected components (LCC). These LCCs were surprisingly similar in size and in diameter but very different in terms of the topological properties if the direction of reactions was taken into account. Interestingly, ACSN reaction graph is almost completely included into one gigantic connected component (Supplementary Figure S7B). REACTOME's LCC was the least dense in terms of the number of reactions. Both LCCs from ACSN and REACTOME are organized into several large strongly connected components and characterized by a wide distribution of directed shortest path lengths. The structure of NCI PID reaction graph is very different: it contains a relatively small core (associated to G-proteins, phosphoinositide-3 kinase pathway and so on), while the rest of the graph is composed from many almost linear and relatively short paths irradiating from or converging to the core (Supplementary Figures S7B and D). This analysis allowed us to conclude that, among the three compared databases, ACSN is characterized by the densest representation of crosstalk and feedback regulations between molecular mechanisms.

We also compared the set of publications used to annotate the three pathway databases. The overlap of ACSN with REACTOME and NCI PID is as small as 10%. ACSN contains relatively more papers published after 2010 than REACTOME and NCI PID (NCI PID was not updated since 2012). The median date of an annotated reference in ACSN is 4 years younger compared with REACTOME and NCI PID (Supplementary Figures S8A and B). ACSN uses reviews (unlike PID which almost never uses review publications) and journals specific to cancer (such as Oncogene and Cancer Research) more frequently and have a relatively broader journal choice than the other pathway databases. For example, 22% of publications used to annotate NCI PID are from *Journal of Biological Chemistry*, while ACSN uses this journal in 13% of annotating references, exploiting other journals more broadly (Supplementary Figure S8C). In general, we concluded that the content of ACSN is not redundant with the two other pathway databases compared, though the most canonical molecular pathways are represented similarly in all of them.

To further quantify the cancer-related specificity of ACSN and its degree of completeness, the coverage of various census lists of cancer-related genes by ACSN was computed. ACSN covers 60% (87 out of 138) of the cancer-driver genes from Vogelstein *et al.*^41^ and 31% (150 out of 488) of the more exploratory Catalogue Of Somatic Mutations In Cancer (COSMIC) census gene list,^42^ demonstrating that ACSN includes a significant number of cancer-related processes for which a sufficient mechanistic understanding exists. ACSN will be extended in the future with new mechanisms such as the role of immunity in cancer.

### Application of ACSN for high-throughput cancer biology data visualization and analysis

#### Types of high-throughput data for visualization and analysis using ACSN

High-throughput data for various cancers are collected at increasing rate, comprising data on gene copy-number variations and mutations, gene expression, miRNA expression, protein quantities, activities, localizations and so on. Data of different nature can be visualized and analysed on top of ACSN maps (Figure 2). The NaviCell web service^43^ can be used to import and visualize expression data (for molecular entities such as mRNAs, proteins, miRNAs), gene copy-number data, mutation data and simple gene lists, such as small interfering RNA (siRNA)-based screening hit list. In a more elaborated situation, a weighted gene list can be visualized such as the distribution of mutation frequencies observed in multiple cancers. The ACSN maps can be used to visualize and interpret the global trends in gene expression data as well as the phosphoproteomic data that are able to provide more relevant information than the gene expression data alone.^44^ Interestingly, users can also visualize the results of any statistical analyses applied to a data set. For instance, a matrix of *P*-values resulting from a differential gene expression analysis between two conditions (for example, cancer/normal) can be visualized by map staining. In addition, users can perform a functional enrichment of ACSN modules statistical analysis from a gene list (for example, a list of differentially expressed genes) directly in the ACSN environment (Supplementary Figure S9). The data visualization and analysis functionality is illustrated in several case studies using cancer data: in the live example of visualization of The Cancer Genome Atlas (TCGA) ovarian cancer data set, in the tutorial, and in the guide available from https://acsn.curie.fr/documentation.html.

#### Visualization of siRNA drug-screening results

High-throughput data analysis generally results in a gene list: for example, a list of differentially expressed genes, the genes that contribute the most to a particular scoring, drug targets or hits of siRNA-based genome-wide screenings, and so on.

RNA interference technologies are often used for identification of genes that drive resistance to drugs.^45^ The results of such studies, especially if they are performed at the genome scale, require network-based analysis.

**Figure 3:**
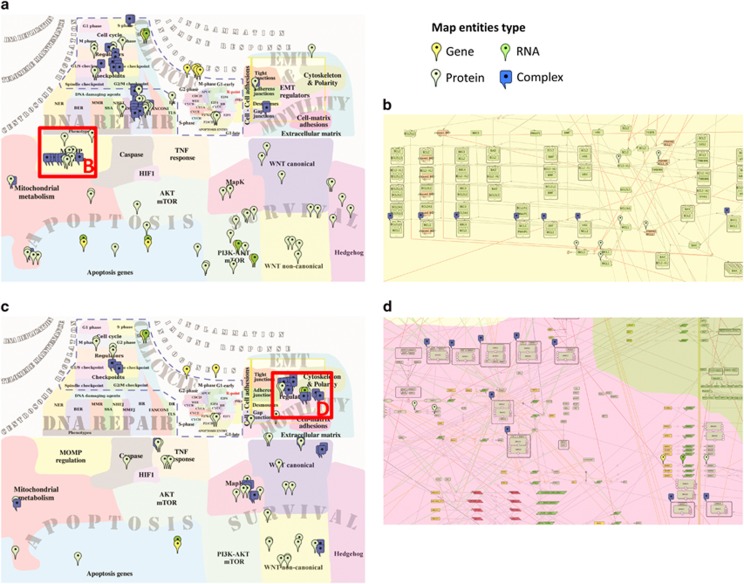
Visualization of siRNA screen hits increasing cell sensitivity to drugs. Visualization of siRNA screen hits increasing cell sensitivity to (**a**) Cisplatin and (**c**) Gemcitabine. Mapping of gene list in ACSN demonstrates coverage across ACSN by the corresponding entities represented by all their molecular modifications as genes, proteins, RNA, complexes and entities with posttranslational modifications. Visualization on the top level zoom shows implication of functional modules. Zooming in to detailed level shows the involved molecular components. (**b**) Molecular modifications of MOMP functional module in Apoptosis map involved in sensitization to Cisplatin. (**d**) Molecular modifications of EMT regulators functional module in EMT and cell motility map involved in sensitization for Gemcitabine.

We have visualized the results of siRNA screens for enhancement of sensitivity to commonly used cancer chemotherapeutics, Cisplatin and Gemcitabine.^46^ We mapped the gene lists of siRNA hits obtained in the study onto ACSN and visualized the distribution of molecular modifications involved in various biological processes (Figure 3 and Table 2). The comparison of processes depicted in ACSN and covered by two siRNA hit lists shows that there are distinct molecular processes involved in the sensitization of cells to each drug, thus indicating different mechanisms of action (Figure 3 and Supplementary Table S2).

Thanks to ACSN's feature distinguishing various molecular modifications, one can visualize the protein complexes that include siRNA hits. Assuming that all components of the complex contribute together to its activity, each individual component of those complexes may be a putative target, in addition to the list of siRNA screen hits that were reported in the original study (Supplementary Table S3).

Furthermore, analysis of functional modules helps to retrieve combinations of molecular mechanisms involved in the sensitization to the drugs. For example, in the original study it was suggested that increase of sensitivity to Cisplatin involves cell cycle checkpoints and double-strand break repair processes, especially homologous recombination. ACSN analysis reveals additional cellular mechanisms affected by Cisplatin such as apoptosis, cell–cell adhesion, mitochondrial metabolism and cell survival. In particular, a local high density of hits can be observed in the mitochondrial outer-membrane potential (MOMP) functional module of the apoptosis map (Figure 3a and Supplementary Table S2). The MOMP functional module depicts the regulation of the intrinsic apoptotic pathway that affects the MOMP. Noticeably, the siRNA hit myeloid cell leukaemia 1 (MCL-1) is involved in numerous complexes in this functional module, sequestering several pro-apoptotic regulators. Thus inhibition of MCL-1 can enhance apoptosis (Figure 3b and Supplementary Table S3), which is in agreement with a recent study showing that suppression of MCL-1 induces mitochondrial-mediated apoptosis and enhances sensitivity to chemotherapy.^47^

In addition to the suggested cell cycle checkpoint and survival pathway-related mechanisms of sensitization to Gemcitabine, unexpectedly high local density of siRNA hits is found in the EMT_REGULATORS functional module. In particular, the siRNA target SMAD4 is found in several molecular complexes together with regulators of EMT (Figure 3c and Supplementary Table S2). It has been shown that overactivation of the EMT machinery may contribute to drug resistance,^48, 49^ making EMT machinery and specifically SMAD4^50^ attractive targets for sensitization to Gemcitabine.

However, it is known that SMAD4 is a multifunctional protein participating in a wide range of cellular processes, such as growth, proliferation, differentiation and apoptosis.^51^ SMAD4 has numerous and often opposite effects on cells and the surrounding tissue in a context-dependent manner. For example, with respect to cancer SMAD4 can act as either suppressor or promoter of tumour progression.^52^ It can exhibit pro-EMT^53, 54^ or anti-EMT and anti-invasive properties^55, 56^ depending on cancer or tissue type, stage of tumour development and microenvironment.^57, 58^

Although inhibition of SMAD4 indeed sensitizes cells to Gemcitabine in the original study, given the involvement of SMAD4 in multiple cellular processes, undesired off-target effects in patients could be anticipated. To avoid these effects, instead of directly inhibiting SMAD4, targeting interactions between SMAD4 and its specific partners might help to drive SMAD signalling towards a less-toxic Gemcitabine-sensitive outcome. Here we suggest that several components of the EMT-related complexes where SMAD4 is found can represent potential targets for sensitization to Gemcitabine (Figure 3d and Supplementary Table S3), thus proposing a rational design for further experiments.

These examples demonstrate that network-based visualization of siRNA screens can help in prioritizing targets and in designing optimal combinations of targets for sensitizing cell to conventional drugs.

#### Visualization of frequently mutated cancer-driver genes in breast and lung cancers

**Figure 4:**
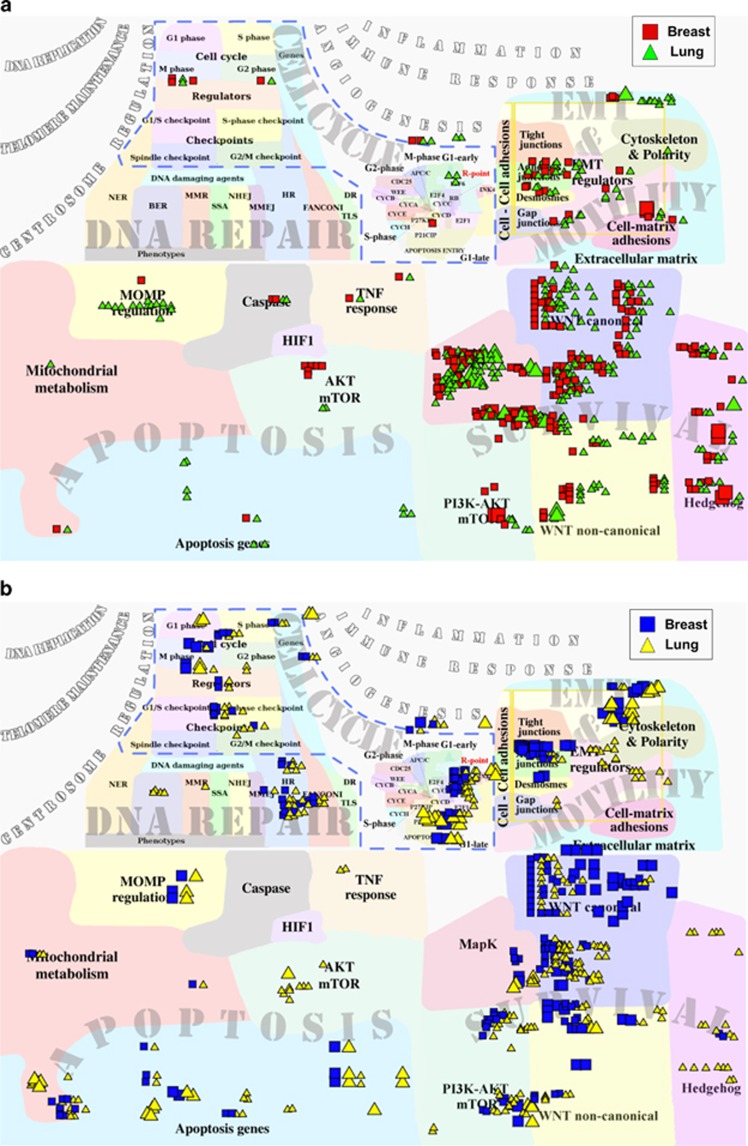
Visualization of ACSN maps coverage by frequently mutated diver genes in breast and lung cancers. Visualization of mutation frequencies in (**a**) oncogenes and (**b**) TSGs in breast and lung cancers. The frequency of a gene mutation is estimated as a percentage of samples carrying this mutation over the number of sample tested in each data set from COSMIC database. The size of the glyphs corresponds to the mutation frequency in the studied data set, varying from high (30–5%), medium (5–1%) to low (1–0.5%).

Cancer progression is associated with the appearance of various mutations. The frequency spectrum of mutations is specific for different types of cancers.^59^ Correlating mutation patterns with other tumour characteristics can help to interpret the role of these mutations in cancer progression. To illustrate applicability of ACSN for visualizing mutation data, we mapped the most frequently mutated oncogenes and tumour-suppressor genes (TSGs) in breast and lung subsets from TCGA data^41^ on ACSN (Figure 4 and Table 3). For both types of cancer, TSG mutations are more frequently found in cell cycle and DNA repair-related modules of ACSN than oncogenes. This might indicate that most probably TSGs in those processes normally contribute to the restriction of uncontrolled divisions and unrepaired DNA but are inactivated by mutations in cancer. Cell survival, EMT and cell motility and cell death related-processes are affected by mutations in both classes of genes in two cancers (Supplementary Table S4). As expected, the coverage of different biological processes by mutated oncogenes and TSGs is not uniform in the two cancer types. Breast cancer (BC) is characterized by the most frequent mutations in oncogenes involved in Hedghog signalling and cell–matrix adhesion, whereas in lung cancer the most frequent oncogene mutations belong to MAPK signalling. The most frequent TSG mutations characterizing BCs are located in WNT canonical pathway and cell–cell adhesion mechanisms. The lung cancer is characterized by high frequency of mutations in apoptosis and mitochondrial metabolism genes (Figure 4 and Supplementary Table S4).

#### Visualization of BC transcriptomes

Interpretation of genome-wide data obtained from cohorts of tumour samples using microarrays or massive sequencing technologies requires functional annotation tools.^60, 61^ For example, visualization of differentially expressed genes on top of biological pathway diagrams may provide insights into the molecular details of cancerogenesis.^39^ ACSN can be used for visualization of both the individual gene expression levels and of the functional module activity level. In our example, the activity of a module is computed by averaging the expression values of participating genes (see Materials and methods). The territory ‘occupied' by a module on the ACSN map is highlighted using a colour gradient denoting the module ‘activity' (see Materials and methods). This ‘map staining' data visualization technique can be combined with other types of data visualization.

**Figure 5:**
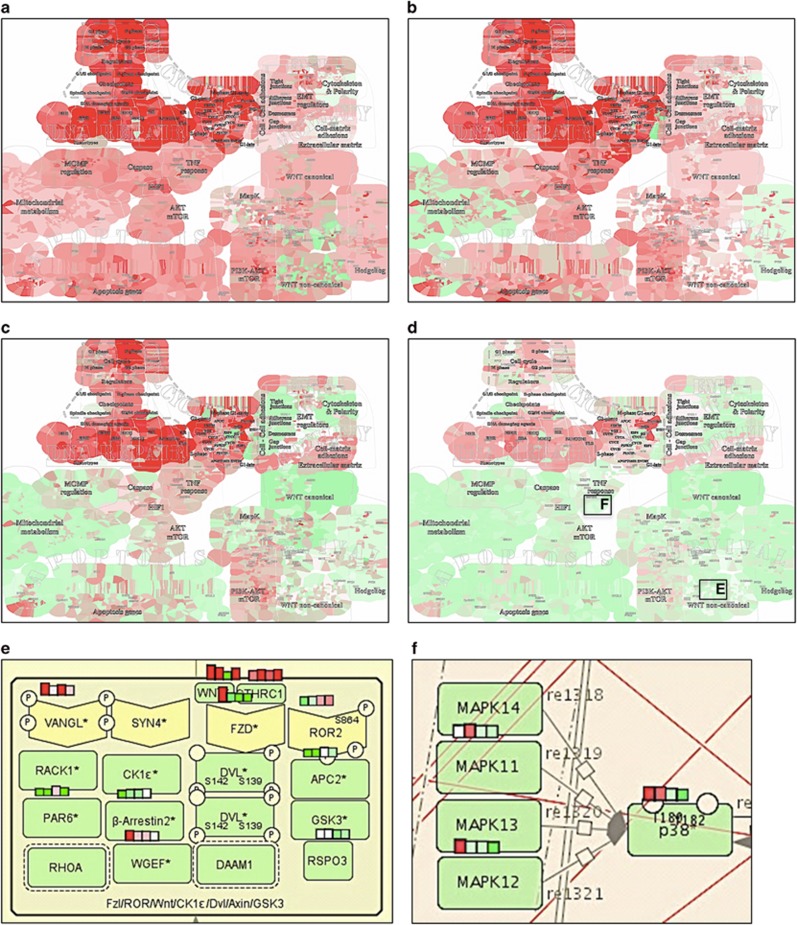
BC gene expression data integration and analysis using ACSN. The mRNA expression data from TCGA collection has been used for evaluation of functional modules activities and ACSN colouring for (**a**) Basal-like, (**b**) Her2-positive, (**c**) Luminal B and (**d**) Luminal A BC types. The four BC subtypes are characterized by different patterns of module activities. Expression values of individual genes for four subtypes of BC are visualized using heat map in NaviCell web-tool. (**e**) Expression levels of genes involved in the same complex for four subtypes of BC. (**f**) Expression levels across member of the gene family for four subtypes of BC. Red colour reflects high expression and green colour shows low expression of the corresponding gene.

To illustrate this approach, mRNA expression data of four BC subtypes from TCGA database were visualized using ACSN. As shown in the Figure 5, the global patterns of ACSN module activities clearly vary between the four BC subtypes. According to the consensus molecular characteristics,^62^ Basal-like tumours are highly mitotic, overexpressing DNA repair-related and phosphoinositide-3 kinase/AKT pathways. Consistently with this, Cell cycle, DNA repair modules and Survival and Apoptosis modules are overactivated in ACSN, (Figure 5a). A practically opposite picture of the activity of ACSN modules is seen in the Luminal A type (Figure 5d); whereas HER2-positive and Luminal B subtypes lie between those two most distinct BC subtypes (Figures 5b and c) that is also consistent with the previous studies.

We also used ACSN to visualize the consensus molecular signatures of BC subtypes.^62^ It is notable that a number of upregulated genes from the Basal-like molecular signature are also involved in functional modules related to the EMT regulators, Cell–Cell adhesions, Cell–Matrix adhesions and Cytoskeletal polarity (Supplementary Figure S10). The activities of the modules EMT regulators and cell–cell adhesions are higher in Basal-like subtype (Figure 5a) compared with Luminal A subtype (Figure 5d). A closer look at the modules demonstrates that in addition to the hits from the consensus molecular Basal-like signature, a number of EMT inducers HIF1, SNAI1, FOXC2 and markers of EMT such as VIM, CDH2, CDH3, KRT5 are also upregulated, but some epithelial markers such as OCLN and CDH1 are downregulated in those modules indicating a higher invasive status in the Basal-like compared with non-invasive Luminal A subtype.^63, 64^ The Cell–Matrix adhesions module has lower activity in Basal-like (Figure 5a) vs Luminal A subtype (Figure 5d). The expression pattern of components of this module points out that numerous collagens and integrins are overexpressed in Luminal A and are lost in Basal-like, which might indicate a rearrangement of cell–matrix connections in Basal-like that may also contribute to invasiveness.^65^ In addition, we visualized mRNA expression levels of each gene represented on ACSN for four BC subtypes simultaneously, allowing, for example, to grasp differences in regulation of genes involved in the same complex (Figure 5e) or differences in expression across members of the gene family (Figure 5f) for four subtypes of BC. Thus various modes of high-throughput data visualization in the context of ACSN help to follow expression changes in individual genes and also to understand the biological functional significance of more general gene expression trends at the level of module activity.

#### Visualization of phosphoproteomic data for lung cancer tumour samples

Although gene expression measurements in tumour samples are the most abundant source of genome-wide data in cancer research, dysfunction of signalling pathways often originates from abnormal regulation of posttranslational modifications in cancer. Recently, qualitative and quantitative data on the posttranslational modifications, especially the phosphorylation status of the key proteins involved in cancer-related signalling, started to be generated at a relatively large scale.^66^

Unlike many existing pathway databases, the representation of molecular events in ACSN includes posttranslational modifications of proteins. When available, ACSN contains information on specific amino-acid modifications. For example, ACSN covers information about 500 phosphorylated protein modifications in addition to other posttranslational modifications. This granularity of representation in ACSN opens the possibility of visualizing protein posttranslational modification data.

**Figure 6:**
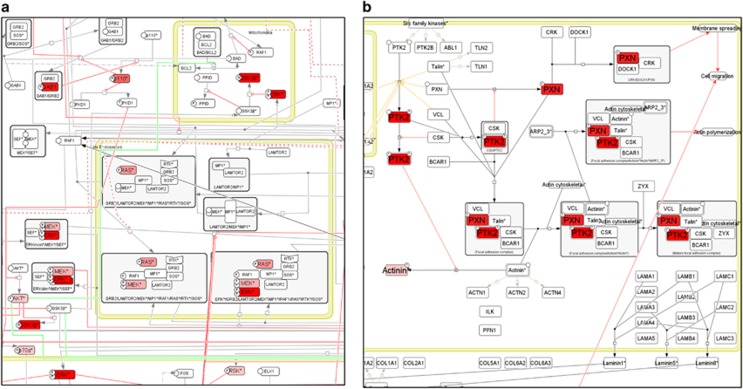
Visualization of phosphoproteomic data using ACSN. The data on phosphorylated forms of proteins in lung cancer samples from the PosphoSitePlus database is mapped onto ACSN maps. The intensity of red colour corresponds to the observed frequency of the phosphorylation event among the tumour samples (pale pink—low to dark red—high frequency). Two regions with relatively high density of phosphorylated protein forms are shown in (**a**) fragment of MAPK module map and (**b**) fragment of cell–matrix adhesion module.

We mapped the frequencies of phosphorylated proteins in lung cancer obtained from the PhosphoSitePlus database^43^ and identified several functional modules on ACSN map where the local density of frequently phosphorylated protein forms was particularly high (Figure 6). Expectedly, the MAPK module is characterized by phosphorylation of classical kinases such as mitogen-activated protein kinase kinase, extracellular signal–regulated kinase, AKT and glycogen synthase kinase 3 beta and their targets (Figure 6a). The role of phosphorylation in the regulation of cell–matrix interactions is a less studied area. Data visualization also sheds light on how phosphorylation events are involved in lung cancer. It is known that phosphorylation of PXN and PKT2 contributes to reorganization of focal adhesions and cell motility in lung cancer.^67^ The interaction between PXN and PTK2 proteins, following their phosphorylation, found in Cell–Matrix adhesion module might be a sign of for increased cell motility potential in the studied lung cancer subset (Figure 6b).

#### Comparison of high-throughput data for visualization and analysis using ACSN and other databases

We have compared the data visualization capabilities in ACSN with other pathway databases (Supplementary Table S1). To demonstrate the different modes of data visualization, we have chosen the maps corresponding to cell cycle processes as the most characterized and detailed cell process found in the majority of pathway databases, including KEGG and REACTOME. As shown in Supplementary Figure S11, ACSN provides a unique possibility to visualize numerous and heterogeneous data types simultaneously, the feature that does not exist in KEGG and REACTOME databases. The data integrated into ACSN maps can be observed at different levels of zoom, from the top level view, where the general pattern of data distribution can be observed, to the most detailed level, where the data associated with individual molecular entities can be studied.

## Discussion

Numerous cell signalling processes and their cross-regulations become aberrant during tumour progression. To understand the crosstalk between different mechanisms and their impact on the disease, we need detailed computer-readable representations of cellular signalling and tools for data analysis on top of them. It is assumed that in pathological situations the structure of a normal cell signalling network is affected by deregulated coordination between pathways or disruption of existing molecular pathways rather than by creating completely new signalling pathways and molecular interactions. The most common abnormalities in pathological situations are perturbations at the level of gene expression, protein abundance or protein posttranslational modifications, irregular ‘firing' or silencing of particular signals, wrong sub-cellular localization of particular molecules and so on.^68, 69^ Such quantitative rather than qualitative network changes compared with normal cell signalling could be studied in the context of comprehensive signalling networks by analysing experimental data obtained from cancer samples, cancer-related cell lines or animal models.

With this aim, we have created ACSN, a web-based resource and environment for systems level study and data analysis in cancer research. It combines a global signalling map representing molecular processes implicated in cancer, a web-based system for navigation, querying and commenting the map and the built-in tools for data visualization and analysis in the context of the signalling map. It is important to underline that ACSN is not a compilation of the information extracted from already existing pathway databases. Although ACSN map managers consult external databases, its content is based on manual literature mining and is, therefore, completely original.

The mechanisms that are depicted in the ACSN maps are generic and reflect the signalling in a normal human cell but that are frequently altered in tumorigenesis. Visualization and analysis of high-throughput data in the context of ACSN allows users to highlight the parts of molecular mechanism deregulated in a particular pathology.

ACSN is a new and actively developed resource. Further extension will include immune response, angiogenesis, telomere maintenance, centrosome regulation, chromatin remodelling mechanisms and so on, which will be integrated into the structure of the Atlas and interconnected with existing maps. With the aim to cover as many molecular processes implicated in cancer as possible and to keep ACSN up-to-date, we anticipate that the maintenance of maps of ACSN maps will become partially a community effort facilitating active discussion and knowledge exchange around these maps via the associated blog system. This paradigm is different from the existing efforts on community-based pathway database creation such as Payao^70^ or WikiPathways.^71^ ACSN updates are controlled by the map managers, applying quality-control procedures and manually introducing the map changes.

The comprehensive maps of molecular interactions may serve for multilevel data integration,^72^ exploiting the concepts used for integrating data on top of geographical maps. Visualizing different data types, from simple gene lists to phosphoproteomic data, is made possible due to the nature of ACSN pathway representation in the form of a global and detailed biochemical reaction network, describing transformations, functions and regulations of different forms of proteins and molecular complexes. This distinguishes ACSN from many other cancer-oriented pathway databases, where molecular mechanisms are depicted at a more coarse-grained level.

In summary, ACSN is a tool that supports transfer of knowledge on molecular mechanisms altered in cancer to the computational methods for diagnosis, prediction of drug resistance, patient stratification and search for synthetic interactions that will help to design combinatorial therapeutic strategies.^73^ Moreover, the signalling processes represented in ACSN are considered to occur in normal mammalian cells: therefore the area of ACSN application is not limited to cancer research.

## Materials and Methods

### ACSN documentation, data and code availability

An introduction into the features of ACSN, video tutorial, downloadable user guide and documentation about ACSN database are available on the ACSN web page (http://acsn.curie.fr). The ACSN maps are freely available for downloading from the ACSN web page (https://acsn.curie.fr/downloads.html) in BioPAX and PNG formats. The module composition of ACSN is available as GMT files. The description of visualization and analysis functionality, including case studies, tutorial, guide and online live examples, are available from https://acsn.curie.fr/documentation.html. The Java code used to assemble the ACSN map and convert it into the NaviCell format is a part of BiNoM Cytoscape plugin and available at http://binom.curie.fr.

### Graphical standards and data model

The knowledge is formalized using Process Description language from the Systems Biology Graphical Notation standard^7^ implemented in the CellDesigner software^8^ (Supplementary Figure S1). ACSN distinguishes the following molecular entities: protein, gene, RNA, antisense RNA, simple molecule, ion, drug, phenotype, complexes. Cellular compartments such as cytoplasm, nucleus, mitochondria, and so on recapitulate the cellular architecture. ACSN is a biochemical reaction network formed by reactants, products and various types of regulators. Edges on the map represent biochemical reactions or reaction regulations of various types, including posttranslational modifications, translation, transcription, complex formation or dissociation, transport, degradation and so on. Reaction regulations include catalysis, inhibition, modulation, trigger and physical stimulation (Supplementary Figure S1). The naming system of ACSN is mostly based on HUGO identifiers for genes, proteins, RNAs and antisense RNAs and CAS identifiers for drugs, small molecules and ions. In some cases, the entities are named by commonly used synonyms.

### ACSN content: literature and database mining and data input

The map curator studies the body of literature dedicated to the biological process or molecular mechanism of interest. The initial sources of information are the major review articles from high-impact journals that represent the consensus view on the studied topic and also provide a list of original references. The map curator extracts information from review papers and represents it in the form of biochemical reactions in CellDesigner. This level of details reflects the ‘canonical' mechanisms. Afterwards, the curator extends the search and analyses original papers from the list provided in the review articles and beyond. This information is used to enrich the map with details from the recent discoveries in the field. The rule for confident acceptance and inclusion of a biochemical reaction or a process is the presence of sufficient evidences from more than two studies, preferably from different scientific groups. The content of ACSN is also verified and compared with publicly available databases such as REACTOME, KEGG, WikiPathways, BioCarta, Cell Signalling and others to ensure comprehensive representation of consensus pathways and links on PMIDs of original articles confirmed annotated molecular interactions.

### ACSN annotation format

Each entity and reaction on the map is annotated using the NaviCell annotation format that includes sections ‘Identifiers', ‘Maps_Modules' and ‘References'. ‘Identifiers' section provides links to the corresponding entity descriptions in HGNC, UniProt, Entrez, GeneCards, REACTOME, Kegg and WikiPathways databases. ‘Maps_Modules' section includes links to maps and modules of ACSN. ‘References' section includes notes added by the map manager and links to relevant publications. Annotations can be associated with a molecular entity (such as protein) as well as to its particular modification (such as a particular posttranslational modification) (Supplementary Figure S3). Each entity on ACSN is also cross-referenced with databases as KEGG PATHWAY,^38^ REACTOME,^74^ Atlas of Genetics and Cytogenetics in Oncology and Haematology,^75^ GeneCards,^76^ Wiki Genes^77^ and others.

### ACSN reaction and protein complex confidence scores

To provide information on the reliability of the depicted molecular interactions, two confidence score have been introduced into ACSN. Both scores represent integer numbers varying from 0 (undefined confidence) to 5 (high confidence). The reference score (REF) indicates both the number and the ‘weight' associated with publications found in the annotation of a given reaction, with weight equal to 1 point for an original publication and 3 points for a review article. For example, a reaction annotated by two reviews will obtain the highest reference score REF=5. The functional proximity score (FUNC) is computed based on the external network of protein–protein interactions (PPI), Human Protein Reference Database, which contains both experimental and literature-based curated interaction data.^78^ The score reflects an average distance in the PPI graph between all proteins participating in the reaction (reactants, products or regulators). Thus, if all proteins participating in a reaction interacts directly (functional distance 1) in the PPI network then this reaction obtains the highest score (FUNC=5). If in the reaction where phosphorylation of protein A is catalysed by protein B, and A and B are separated in PPI graph by 2 (or 3, or 4) edges, then the reaction obtains the functional proximity score FUNC=4 (or FUNC=3, or FUNC=2 correspondingly). If two reaction participants are not connected in the PPI network, then FUNC=0. The functional proximity is computed using BiNoM Cytoscape plugin.^39, 40^ Both scores are visualized in the ‘Confidence' section of the ACSN annotations as a coloured ‘five-star' diagram (Supplementary Figure S3). Same scores are assigned to each protein complex in ACSN, with the same calculations performed for the protein complex components.

### ACSN curation and commenting

Each entity annotation is represented as a post in the web-blog providing a possibility of communication between map users and map managers. Any user can submit comments in the form of hyperlinked text together with files of any type, if needed (Supplementary Figures S2 and S3).

### High-throughput data sources and analysis

To illustrate the coverage of gene mutation frequencies over the ACSN maps, the information about mutations in breast and lung cancers was obtained from the COSMIC database.^42^ The frequency of a gene mutation is represented as a ratio of samples carrying a mutation over the number of samples tested in each data set.

For mRNA expression analysis, normalized RNASeq data for 745 breast tumours and 100 healthy samples were obtained from TCGA database. Raw count data were first normalized using the Upper Quartile method^79^ and then grouped into the four BC subtypes:^80^ Basal-like (79 samples), Her2-positive (32 samples), Luminal A (385 samples), and Luminal B (118 samples). Each gene was assigned a score using median expression level across samples of the same subgroup, for ACSN map staining. Each functional ACSN module was attributed a score based on the average score of its genes. This score was visualized using BiNoM.^39, 40^

For phosphoproteomic analysis, the phosphorylation data for a cohort of 234 lung tumour samples were obtained from PhosphoSitePlus database.^43^ About 130 phosphorylated protein forms from the data set were found on the ACSN maps. If ACSN contained only a generic representation of a ‘phosphorylated' protein, then all residue frequencies related to this protein were summed up. Visualization of data was performed using BiNoM.^39, 40^

### Data visualization and analysis in ACSN using web-based NaviCell environment

ACSN uses NaviCell environment to represent the content of the ACSN map online (Kuperstein *et al.*^36^). NaviCell includes a panel of tools for visualization and analysis of high-throughput data (Figure 2). The current version of the tool box allows to load users' data such as gene lists, copy-number profiles, transcriptome or protein expression data sets and to visualize them on top of ACSN map. The visualization techniques can be configured by the user and include bars and heatmaps or glyphs located near each molecular entity as well as original methods of ‘map staining' for visualizing transcriptome, proteome or copy-number data sets, displaying the data at the background of the map. The data visualization modes can be combined in multiple ways, providing flexibility in representing several data types simultaneously at several zoom levels of the map. The data can be visualized for separate samples or for groups of samples. These groups can be defined by the user according to a selected sample feature (for example, molecular tumour subtype), and used further in data visualization. All functions of NaviCell and ACSN can be programmatically manipulated through NaviCell web service,^43^ using Python or R languages.

In ACSN, users have the possibility to perform the most frequently used types of functional data analysis, which exploit the content of ACSN pathway database, such as functional enrichment of ACSN modules in a gene list. A hypergeometric test can be performed to compute the *P*-values of intersections of a given gene list with ACSN modules (see Supplementary Figure S9).

## Figures and Tables

**Table 1:** Content of ACSN maps and modules

*Map/module*	*Modules*	*Chemical species*	*Proteins*	*Reactions*	*References*	*Creation date*	*Last update*
Apoptosis map	7	1640	687	1166	595	2010	2014
Cell cycle map	17	165	78	165	235	2008	2011
Cell survival map	5	1927	555	1305	846	2011	2015
DNA repair map	18	733	377	545	635	2010	2014
EMT and cell motility map	5	1510	674	1645	608	2012	2014
ACSN global map	52	5975	2371	4826	2919	2013	2015

Abbreviations: ACSN, Atlas of Cancer Signalling Network; EMT, epithelial-to-mesenchymal transition.

**Table 2:** Coverage of ACSN maps and modules with siRNA screen hits increasing sensitivity for Cisplatin and Gemcitabine

*Drug*	*siRNA hits*	*In ACSN*	*Number of found ACSN molecular species*	*Number of maps and modules containing the hits*
Cisplatin	37	13	123 (complexes 34)	5/21
Gemcitabine	24	10	77 (complexes 21)	5/12

Abbreviations: ACSN, Atlas of Cancer Signalling Network; siRNA, small interfering RNA.

**Table 3:** Coverage of ACSN maps and modules with frequent mutations in oncogenes and tumour-suppressor genes in breast and lung cancers

*Gene status*	*Total list*	*In ACSN*	*Number of found ACSN molecular species*	*Number of maps and modules containing the mutated genes*
*Breast cancer*				
Oncogenes	29	18	113	5/27
TSGs	39	35	336	5/42
				
*Lung cancer*				
Oncogenes	29	29	180	5/25
TSGs	39	39	378	5/42

Abbreviations: ACSN, Atlas of Cancer Signalling Network; TSG, tumour-suppressor gene.
