# Supplementary material for: Atlas of Cancer Signalling Network: a systems biology resource for integrative analysis of cancer data with Google Maps
Source: Oncogenesis. 2015 Jul 20;4(7):e160–. doi: 10.1038/oncsis.2015.19 (PMC4521180; doi:10.1038/oncsis.2015.19)
Supplement: Supplementary Tables and Figure Legends [file oncsis201519x1.docx]

**Supplementary Table 1**

Comparison of ACSN to existing pathways databases

|  | **Database characteristics** | | | | | **Navigation** | | | **Data**  **visualization and analysis** | | |
| --- | --- | --- | --- | --- | --- | --- | --- | --- | --- | --- | --- |
| **Database** | **Web link** | **Exchange formats** | **SBGN support** | **Release**  **year** | **Overlap with ACSN**  **(Functions)** | **Zoom** | **Modular structure** | **Possibility to comment on the content** | **Built in data visualization tool** | **Built in functional analysis** | **Built in path finding analysis** |
| **REACTOME** | [http://www.REACTOME.org](http://www.reactome.org) | BioPAX SBML PDF Word | ● | 2005 | Cell-cell communication; apoptosis; cell cycle; pathways related to survival and DNA repair | ●* | ● |  | ● | ● | ● |
| **KEGG PATHWAY** | <http://www.genome.jp/pub/kegg> | BioPAX SBML KGML | ● | 1995 | Cell adhesion; apoptosis; cell cycle; DNA repair; pathways related to survival | ● |  |  | ● |  |  |
| **WikiPathways** | <http://www.wikipathways.org> | GPML SBML BioPAX PDF SVG |  | 2008 | Apoptosis, Cell cycle, pathways related to survival and DNA repair | ● |  | ● |  |  |  |
| **NCI** | <http://pid.nci.nih.gov> | XML BioPAX JPG SVG |  | 2005 | Pathways related to apoptosis, cell cycle regulation, pathways related to survival, pathways related to DNA repair, cell adhesion | ● |  |  |  |  |  |
| **SPIKE** | <http://www.cs.tau.ac.il/~spike> | SBML BioPAX SIF |  | 2008 | Apoptosis, cell cycle, pathways related to survival and DNA repair | ● |  |  |  |  |  |
| **ACSN** | <https://acsn.curie.fr> | BioPAX GMT PNG | ● | 2013 |  | ●* | ● | ● | ●** | ● |  |

*Semantic zoom

**Single and multiple data types

**Supplementary Table 2**

Coverage of ACSN molecular species across maps and modules by siRNA hits from drug sensitivity study for Cisplatin and Gemcitabine. Genes coverage column shows how many protein names from the hit list are found in a module. Molecular species coverage column shows how many molecular modifications (post-translational, complexes, different localisations) correspond to this number of genes in the module. The p-values are computed using the standard hypergeometric test. If a protein is represented on the map by many modifications, it points out to its important role in the process (weight) and the p-values are calculated accordingly.

| ***Drug*** | ***Map*** | ***Module*** | ***Genes***  ***coverage*** | ***p- value*** | ***Molecular species coverage*** | ***p- value*** |
| --- | --- | --- | --- | --- | --- | --- |
| Cisplatin | DNA repair | S phase | 3 | 7.7E-02 | 2 | 5.73E-01 |
|  |  | G1/S checkpoint | 3 | 1.2E-02 | 1 | 6.95E-01 |
|  |  | S phase checkpoint | 3 | 1.9E-02 | 7 | 1.50E-03 |
|  |  | Spindle checkpoint | 1 | 3.7E-01 | 2 | 2.11E-01 |
|  |  | A_NHEJ | 3 | 6.6E-03 | 2 | 2.39E-04 |
|  |  | C_NHEJ | 4 | 1.8E-03 | 6 | 7.87E-19 |
|  |  | HR | 7 | 3.9E-06 | 23 | 4.67E-01 |
|  |  | TLS | 1 | 2.1E-01 | 1 | 5.33E-02 |
|  | Cell Cycle | Apoptosis Entry | 2 | 1.1E-02 | 3 | 4.46E-05 |
|  | Apoptosis | MOMP regulation | 4 | 2.4E-02 | 16 | 7.13E-01 |
|  |  | Mitochondrial metabolism | 5 | 3.7E-01 | 9 | 6.19E-01 |
|  |  | Apoptosis genes | 4 | 1.4E-01 | 9 | 9.45E-01 |
|  |  | AKT-mTOR | 2 | 1.5E-01 | 1 | 1.71E-01 |
|  | Cell survival | PI3K-AKT-mTOR | 6 | 4.4E-02 | 11 | 7.84E-01 |
|  |  | WNT non-canonical | 4 | 5.6E-01 | 6 | 9.96E-01 |
|  |  | WNT canonical | 3 | 7.8E-01 | 3 | 9.12E-01 |
|  |  | MAPK | 1 | 8.6E-01 | 3 | 8.08E-01 |
|  |  | Hedgehog | 2 | 7.6E-01 | 5 | 5.73E-01 |
|  | EMT and motility | Adherent junctions | 1 | 8.3E-01 | 2 | NA |
|  |  | Gap junctions | 1 | NA | 2 | NA |

| Gemcitabine | DNA repair | S phase checkpoint | 1 | 2.6E-01 | 3 | 7.59E-02 |
| --- | --- | --- | --- | --- | --- | --- |
|  | Cell Cycle | Apoptosis Entry | 1 | 7.8E-02 | 2 | 8.71E-02 |
|  | Apoptosis | Caspase | 2 | 9.1E-02 | 1 | 8.73E-01 |
|  |  | Mitochondrial metabolism | 2 | 6.2E-01 | 3 | 9.44E-01 |
|  |  | Apoptosis genes | 2 | 2.8E-01 | 3 | 9.21E-01 |
|  |  | TNF response | 2 | 7.7E-02 | 7 | 1.13E-02 |
|  | Cell survival | WNT non-canonical | 3 | 3.0e-01 | 11 | 3.91E-03 |
|  |  | WNT canonical | 1 | 8.9e-01 | 10 | 3.47E-02 |
|  |  | MAPK | 3 | 6.0e-02 | 10 | 9.32E-04 |
|  | EMT and motility | EMT regulators | 2 | 9.3e-02 | 19 | 2.57E-09 |

Supplementary Table 3

Coverage of protein complexes by siRNA hits hits from drug sensitivity study for cisplatin and Gemcitabine

| ***Drug*** | ***Complexes on ACSN containing siRNA hits*** |
| --- | --- |
| Cisplatin | ACACA:BRCA1  ATR:ATRIP  ATR:ATRIP:CHEK1:CLSPN:HCLK2*  ATR:ATRIP:CHEK1:CLSPN:PCNA:TIM1*  ATR:ATRIP:CHEK1:CLSPN:RPA1:RPA2:RPA3:TIM1*:TIPIN  ATR:ATRIP:CLSPN:RAD17:TOPBP1  ATR:ATRIP:CLSPN:RPA1:RPA2:RPA3  ATR:FAAP24*:FANCM:HCLK2*  ATR:FANCD2:MRE11*:NBS1*:RAD50  AURCA:BRCA1  AXIN1:DVL1:FRAT1  BAK1:MCL1  BARD1:BRCA1  BARD1:BRCA1:BRCA2:BRIT1*:DSS1*:FANCD2:FANCN*:RAD51:RAD52:SFPQ:XRCC2:XRCC3  BARD1:BRCA1:BRCC3:FAM175A:UIMC1  BBC3:MCL1  BCL2L11:MCL1  BMF:MCL1  BRCA1:BRCA2:FANCD2:FANCI:FANCJ*:FANCN*:RPA1:RPA2:RPA3  BRCA1:BRCA2:FANCD2:FANCI:H2AFX:NBS1*:PCNA:RAD51:TIP60*  BRCA1:CTIP*  BRCA1:CTIP*:MRE11*:NBS1*:PARP1:PARP2:RAD50  BRCA1:E2F1:RB1  BRCA1:FANCJ*  BRCA1:SMC1*  BRCA1:SMC3  BRCA2:DSS1*  BRCA2:FANCC:FANCG:XRCC3  HRK:MCL1  MCL1:PMAIP1  MCL1:cleaved_BID*  MIR34A:NOTCH1  MIR449A:NOTCH1  RAD51:XRCC2:XRCC3 |
| Gemcitabine | ATR:ATRIP:CHEK1:CLSPN:HCLK2*  ATR:ATRIP:CHEK1:CLSPN:PCNA:TIM1*  ATR:ATRIP:CHEK1:CLSPN:RPA1:RPA2:RPA3:TIM1*:TIPIN  CFLAR:FADD:RIPK1:TRADD  CSE1L:Ca2+:Calmodulin*:KPNB1:LRRK2:NFAT*:NRON:PPP3CB:PPP3R*:TNPO1:Tubulin  CSE1L:KPNB1:LRRK2:NFAT*:NRON:PPP3CB: PPP3R*:TNPO1:Tubulin  DVL2:LRP6:RIPK4  DVL2:NKD1  DVL2:RIPK4  Fe2+:RRM1:RRM2*  GRB2:MAP3K1:RAS*:RTK*:SOS*  MAP3K1:RIPK1:TNFRSF1A:TRADD:TRAF2  MAP3K1:TNFRSF1B:TRAF2  MIZ1*:SMAD2:SMAD3:SMAD4:SP1  SMAD2:SMAD3:SMAD4  SMAD2:SMAD3:SMAD4:SP1  SMAD2:SMAD3:SMAD4:ZEB1  SMAD2:SMAD4  SMAD3:SMAD4  SMAD3:SMAD4:SNAI1  SMAD3:SMAD4:SP1 |

The element of complexes from siRNA screen is marked by blue

Supplementary Table 4

Coverage of ACSN elements across maps and modules by frequently mutated oncogenes and tumour suppressor genes in Breast and Lung cancers. The p-values are computed using the standard hypergeometric test.

| ***Oncogenes*** |  | ***Breast cancer*** | | | | ***Lung cancer*** | | | |
| --- | --- | --- | --- | --- | --- | --- | --- | --- | --- |
| ***Map*** | ***Module*** | ***Genes***  ***coverage*** | ***p- value*** | ***Molecular species coverage*** | ***p- value*** | ***Genes***  ***coverage*** | ***p- value*** | ***Molecular species coverage*** | ***p- value*** |
| DNA repair |  |  |  | 4 |  |  |  | 3 |  |
|  | G1 phase | 1 | 3.9E-01 | 1 | 6,30E−01 | 1 | 5.7e-01 | 1 | 7,83E−01 |
|  | S phase | 1 | 5.7E-01 | 1 | 8,24E−01 | 1 | 7.6e-01 | 1 | 9,31E−01 |
|  | G2_M checkpoint | 1 | 1.1E-01 | 3 | 1,98E−01 | 2 | 2.5e-01 | 3 | 5,19E−01 |
|  | G1_S checkpoint | 1 | 3.3e-01 | 1 | 6,54E−01 |  |  | 0 |  |
|  | C_NHEJ | 1 | 3.6e-01 | 1 | 5,60E−01 | 1 | 5.3e-01 | 1 | 7,17E−01 |
|  | S phase checkpoint | 2 | 7.8e-02 | 3 | 1,98E−01 | 2 | 1.9e-01 | 3 | 4,17E−01 |
|  | Regulators | 2 | NA | 4 | NA | 2 | NA | 3 | NA |
| Cell Cycle |  |  |  | 1 |  |  |  | 2 |  |
|  | Apoptosis Entry | 1 | 1.2e-01 | 1 | 5,41E−01 |  |  | 0 |  |
|  | E2F6 |  |  | 0 |  | 1 | 2.0e-01 | 3 | 1,48E−01 |
| Apoptosis |  |  |  | 11 |  |  |  | 44 |  |
|  | MOMP regulation | 1 | 6.1e-01 | 4 | 6,83E−01 | 1 | 8.0e-01 | 33 | 2,41E−14 |
|  | Mitochondrial metabolism | 2 | 8.6e-01 | 2 | 9,99E−01 | 3 | 9.3e-01 | 32 | 6,30E−06 |
|  | Apoptosis genes |  |  | 0 |  | 2 | 7.9e-01 | 5 | 9,97E−01 |
|  | AKT-mTOR | 2 | 1.1e-01 | 7 | 1,30E−02 | 2 | 2.5e-01 | 6 | 1,90E−01 |
|  | Caspases | 2 | 2.0e-01 | 6 | 9,26E−02 | 2 | 4.1e-01 | 16 | 1,85E−05 |
|  | TNF response | 1 | 5.4e-01 | 1 | 9,81E−01 | 2 | 3.6e-01 | 3 | 9,38E−01 |
| Cell survival |  |  |  | 81 |  |  |  | 91 |  |
|  | PI3K-AKT-mTOR | 10 | 7.0e-06 | 14 | 1,08E−02 | 12 | 8.2e-05 | 12 | 4,04E−01 |
|  | WNT non-canonical | 8 | 5.4e-03 | 21 | 2,67E−06 | 11 | 8.0e-03 | 24 | 8,79E−05 |
|  | WNT canonical | 2 | 8.5e-01 | 14 | 3,11E−02 | 4 | 8.0e-01 | 18 | 6,97E−02 |
|  | MAPK | 10 | 2.2e-07 | 14 | 2,75E−04 | 13 | 2.0e-07 | 17 | 9,76E−04 |
|  | Hedgehog | 7 | 2.5e-03 | 18 | 2,14E−05 | 9 | 5.5e-03 | 20 | 7,04E−04 |
| EMT and motility |  |  |  | 35 |  |  |  | 62 |  |
|  | Cell-Cell adhesions | 2 | 4.0e-01 | 5 | 5,89E−01 | 3 | 4.1e-01 | 6 | 8,05E−01 |
|  | ECM | 2 | 3.2e-01 | 2 | 8,91E−01 | 2 | 5.8e-01 | 2 | 9,80E−01 |
|  | Cell-Matrix adhesions | 1 | 4.5e-01 | 1 | 9,86E−01 | 1 | 6.4e-01 | 1 | 9,99E−01 |
|  | Cytoskeleton&polarity |  |  | 0 |  | 2 | 6.5e-01 | 2 | 9,97E−01 |
|  | EMT regulators | 4 | 8.0e-03 | 5 | 7,09E−01 | 4 | 4.9e-02 | 10 | 4,10E−01 |

| ***TSGs*** |  | ***Breast cancer*** | | | | ***Lung cancer*** | | | |
| --- | --- | --- | --- | --- | --- | --- | --- | --- | --- |
| ***Map*** | ***Module*** | ***Genes coverage*** | ***p- value*** | ***Molecular species coverage*** | ***p- value*** | ***Genes coverage*** | ***p- value*** | ***Molecular species coverage*** | ***p- value*** |
| DNA repair |  |  |  | 66 |  |  |  | 66 |  |
|  | G1 phase | 4 | 1.9E-02 | 12 | 1,60E-05 | 4 | 2.7E-02 | 12 | 2,32E-05 |
|  | S phase | 3 | 2.4E-01 | 11 | 1,02E-02 | 3 | 3.0E-01 | 11 | 1,32E-02 |
|  | M phase | 4 | 3.7E-03 | 8 | 3,27E-03 | 4 | 5.6E-03 | 8 | 4,10E-03 |
|  | BER | 4 | 2.7E-02 | 13 | 1,03E-04 | 4 | 3.9E-02 | 13 | 1,50E-04 |
|  | MMR | 4 | 6.5E-03 | 13 | 1,26E-08 | 4 | 9.6E-03 | 13 | 1,97E-08 |
|  | SSA | 1 | 3.7E-01 | 4 | 1,17E-02 |  |  |  |  |
|  | A_NHEJ | 2 | 1.5E-01 | 7 | 5,97E-03 | 2 | 1.7E-01 | 7 | 7,27E-03 |
|  | HR | 3 | 1.3E-01 | 21 | 3,60E-08 | 3 | 1.6E-01 | 21 | 6,83E-08 |
|  | Fanconi | 5 | 7.3E-03 | 20 | 4,09E-09 | 5 | 1.2E-02 | 20 | 7,73E-09 |
|  | G2_M checkpoint | 6 | 9.6E-04 | 19 | 5,97E-07 | 6 | 1.8E-03 | 19 | 1,05E-06 |
|  | G1_S checkpoint | 8 | 8.2E-07 | 17 | 2,25E-09 | 8 | 2.0E-06 | 17 | 3,95E-09 |
|  | C_NHEJ | 5 | 2.0E-03 | 7 | 8,10E-03 | 5 | 3.3E-03 | 7 | 9,84E-03 |
|  | S phase checkpoint | 2 | 2.6E-01 | 12 | 1.45E-03 | 2 | 3.1E-01 | 12 | 1.99E-03 |
| Cell Cycle |  |  |  | 41 |  |  |  | 41 |  |
|  | Apoptosis Entry | 2 | 3.0E-02 | 10 | 4,58E−05 | 2 | 3.7E-02 | 10 | 6,26E-05 |
|  | RB | 2 | 5.9E-02 | 11 | 5,13E−11 | 2 | 7.2E-02 | 11 | 7,66E-11 |
|  | E2F1 | 5 | 1.7E-05 | 9 | 2,85E−03 | 5 | 3.0E-05 | 9 | 3,64E-03 |
|  | INK4 | 1 | 7.4E-02 | 1 | 1,21E−01 | 1 | 8.3E-02 | 1 | 1,25E-01 |
| Apoptosis |  |  |  | 49 |  |  |  | 57 |  |
|  | MOMP regulation | 3 | 3.1E-01 | 7 | 9,85E-01 | 3 | 3.7E-01 | 7 | 9,89E-01 |
|  | Mitochondrial metabolism | 2 | 1.0E+00 | 14 | 9,99E-01 | 3 | 9.9E-01 | 15 | 9,98E-01 |
|  | Apoptosis genes | 7 | 5.4E-02 | 21 | 8,27E-01 | 7 | 9.0E-02 | 21 | 8,72E-01 |
|  | AKT-mTOR | 1 | 7.1E-01 | 8 | 4,66E-01 | 2 | 3.9E-01 | 15 | 9,25E-03 |
|  | Caspases | 1 | 8.4E-01 | 3 | 9,96E-01 | 2 | 6.0E-01 | 4 | 9,88E-01 |
|  | TNF response | 2 | 4.8E-01 | 5 | 9,90E-01 | 3 | 2.6E-01 | 6 | 9,80E-01 |
|  | HIF1 | 2 | 7.6E-02 | 4 | 1,97E-01 | 2 | 9.2E-02 | 4 | 2,15E-01 |
| Cell survival |  |  |  | 150 |  |  |  | 154 |  |
|  | PI3K-AKT-mTOR | 7 | 1.7E-01 | 31 | 1,53E-02 | 8 | 1.3E-01 | 33 | 8,63E-03 |
|  | WNT non-canonical | 7 | 4.9E-01 | 20 | 5,31E-01 | 9 | 3.0E-01 | 22 | 4,14E-01 |
|  | WNT canonical | 14 | 2.7E-03 | 74 | 3,74E-20 | 14 | 8.9E-03 | 74 | 3,69E-19 |
|  | MAPK | 6 | 9.4E-02 | 14 | 5,52E-01 | 6 | 1.4E-01 | 14 | 6,09E-01 |
|  | Hedgehog | 7 | 1.5E-01 | 11 | 9,70E-01 | 7 | 2.2E-01 | 11 | 9,79E-01 |
| EMT and motility |  |  |  | 75 |  |  |  | 75 |  |
|  | Cell-Cell adhesions | 5 | 1.6E-01 | 21 | 7,27E-02 | 5 | 2.2e-01 | 21 | 9,75E-02 |
|  | ECM | 3 | 4.4E-01 | 3 | 9,99E-01 | 3 | 5.1e-01 | 3 | 9,99E-01 |
|  | EMT regulators | 9 | 3.8E-05 | 40 | 2,76E-07 | 9 | 9.8e-05 | 40 | 7,36E-07 |

**Supplementary figure legends**

**Supplementary Figure 1. ACSN data model**

The scheme depicts typical entities; most common types of reactions and regulators; cell compartments and transport of entities between them. Symbols and style are almost entirely borrowed from CellDesigner software’s notation, as this was the environment used for building ACSN maps.

**Supplementary Figure 2. ACSN blog system via NaviCell tool: molecular entity annotation post**

Post of MYC protein providing common IDs, links to external databases, ACSN Maps and Modules where the protein appears, corresponding references in PubMed and list of clickable reactions where the entity participates.

**Supplementary Figure 3. ACSN blog system via NaviCell tool: reaction annotation post and confidence score**

The number of filled stars shows how many articles confirm the interaction (one experimental article = one filled star, one review = three filled stars). The background star color corresponds to the confidence score computed based on the average functional distance between interacting proteins calculated from HPRD (Human Protein Reference Database, http://www.hprd.org/) curated protein-protein interaction network. The star color changes from grey/black. Value «0» is assigned to the reactions for which the confidence cannot be computed (such that self-interacting proteins, transport reactions, etc); through green (value «3» means that interaction between the proteins is indirect and mediated by other proteins) to rose/red (value «5» corresponds to direct physical contact of macromolecules, as it is documented in the HPRD interaction network). (B) Comparison of distributions of functional confidence values in ACSN versus randomly selected protein sets.

**Supplementary Figure 4. ACSN semantic zoom levels**

(A) The top-level view shows the general architecture of the atlas, (B). Location of known oncogenes or cancer suppressor genes are visualized, (C) The most participating proteins and complexes in the atlas are visualized, (D), (E) All components and reaction edges between them are visualized, (F) All details of maps are shown including names of all entities, post-translational modifications, names of complexes, reaction identifiers and reaction regulators.

**Supplementary Figure 5. Map pruning for canonical pathways demonstration**

The complexity reduction of the MAPK module in Survival map and of the Cell Cycle map in ACSN has been performed by manual map pruning using the content of the corresponding pathways from REACTOME and KEGG PATHWAY databases. (A) Detailed MAPK module map, (B) Pruned MAPK module map, (C) Detailed Cell Cycle map, (D) Pruned Cell Cycle map.

**Supplementary Figure 6. Exploring neighborhood on the reaction graph**

Right-clicking on the molecular species of interest (RB1) opens a contextual menu allowing to highlight the species, to center the map on the species and to highlight the neighbours of the species on the reaction graph. The function ‘Select and Highlight Neighbours’ highlights all reactions in which a molecular species is involved, as well as all participants of these reactions. (A) Molecular species and reactions connected to the species of interest (RB1) in the reaction graph. (B) Applying the action to any highlighed species allows expanding the neighbourhood of the species of interest (RB1). (C) The function allows highlighting «distantly» located reactants interacting with a molecular species of interest (GSK3β*).

**Supplementary Figure 7. Comparison of ACSN to other databases for the molecular information density**

(A) Comparison of basic properties of ACSN compared to REACTOME and National Cancer Institute Pathway Interaction Database (NCI PID). The ratio between reactions and proteins is computed by only considering reactions involving at least one protein (which excludes purely metabolic reactions in REACTOME and in NCI PID), and proteins explicitly participating to at least one reaction.

For enumeration of complexes, all protein complex modifications are taken into account, including complexes of protein complexes for REACTOME. The maximal values of features are underlined. (B) Hairball visualizations of reaction graph decompositions into connected components, using organic Cytoscape layout. The reaction graphs are extracted from the corresponding BioPAX files with use of BiNoM plugin of Cytoscape. In all three cases, small molecules were eliminated from the graph as well as the node representing ubiquitin at cytosol in REACTOME whose presence largely affected the graph connectivity properties of the praph. Properties of the largest connected component of the reaction graph (LCC) are indicated below it, and were computed using NetworkAnalysis Cytoscape plugin. Characteristic path length (the most probable length of the shortest path) is computed separately for the case when the reaction graph is considered directed and undirected. (C) Distribution of directed path length across each one of the three reaction graph LCC. (D) Both REACTOME and ACSN (but not NCI PID) reaction graph LCCs contain three large (>100 nodes) strongly connected components (SCC). The fraction of LCC covered by each SCC is demonstrated for the three graphs.

**Supplementary Figure 8. Comparison of REACTOME, NCI PID and ACSN pathway databases based on referenced publications**

(A) Distribution of the age of the publications in ACSN compared to REACTOME and National Cancer Institute Pathway Interaction Database (NCI PID). (B) Venn diagram showing intersection of the publications used to construct the three different pathway databases. (C) Relative use of different journals for annotating pathway databases. The journals which are used in ACSN more frequently compared to the other two databases are indicated by arrows. Use of Journal of Biological Chemistry is shown by out of scale numbers. On the right, the total number of different journals is shown used to annotate ASCN, REACTOME and NCI PID respectively, together with the same numbers divided by the total number of publication references contained in the databases.

**Supplementary Figure 9. Gene enrichment analysis using ACSN functional modules**

The list of genes most contributing to one of the Independent Components (CIT7) calculated for bladder cancer expression data (for details of this analysis, see Biton et al, 2014, Cell Rep. 9(4):1235-45) has been used for the enrichment analysis. First column: ACSN functional module’s name, second column: number of unique genes in the module, third column: : number of module’s genes in the component, fourth column: p-value obtained through hypergeometric test, fifth column: list of module’s genes (HUGO IDs) in the component.

**Supplementary Figure 10. Visualization of the signature for Basal-like upregulated genes**

Selection and visualization of the upregulated genes among the consensus molecular signatures for Basal-like breast cancer as reported in The Cancer Genome Atlas Network, Comprehensive molecular portraits of human breast tumours. Nature 490: 61-70 (2012).

**Supplementary Figure 11. Comparison of transcriptome data visualization on top of the cell cycle-related maps from KEGG, REACTOME and ACSN**

The TCGA high-throughput data, ovarian cancer (OVCA) dataset, was used for data visualization, using three data visualization tools. We have computed the fold change statistics values for comparing Proliferative vs. Mesenchymal molecular subtypes of ovarian cancer. Therefore, upregulation of the cell cycle genes is expected. (A) Visualization of three types of data in the context of ACSN at different zoom levels: global ACSN map (left panel); cell cycle map (middle panel), part of G1 cell cycle phase (right panel). Map staining (mRNA expression data, Proliferative/Mesenchymal fold change values); bar plot (copy number data); glyph (mutation frequency data) were used to represent different types of data. Visualization of mRNA expression data (Proliferative/Mesenchymal fold change values) in the context of (B) KEGG cell cycle map and (C) REACTOME G2, G2-M cell cycle phase and S cell cycle phase maps. Upregulation of cell cycle genes is clearly seen in ACSN visualization but less clear in other databases.
