# Supplementary figures and images for: Atlas of Cancer Signalling Network: a systems biology resource for integrative analysis of cancer data with Google Maps
Source: Oncogenesis. 2015 Jul 20;4(7):e160–. doi: 10.1038/oncsis.2015.19 (PMC4521180; doi:10.1038/oncsis.2015.19)

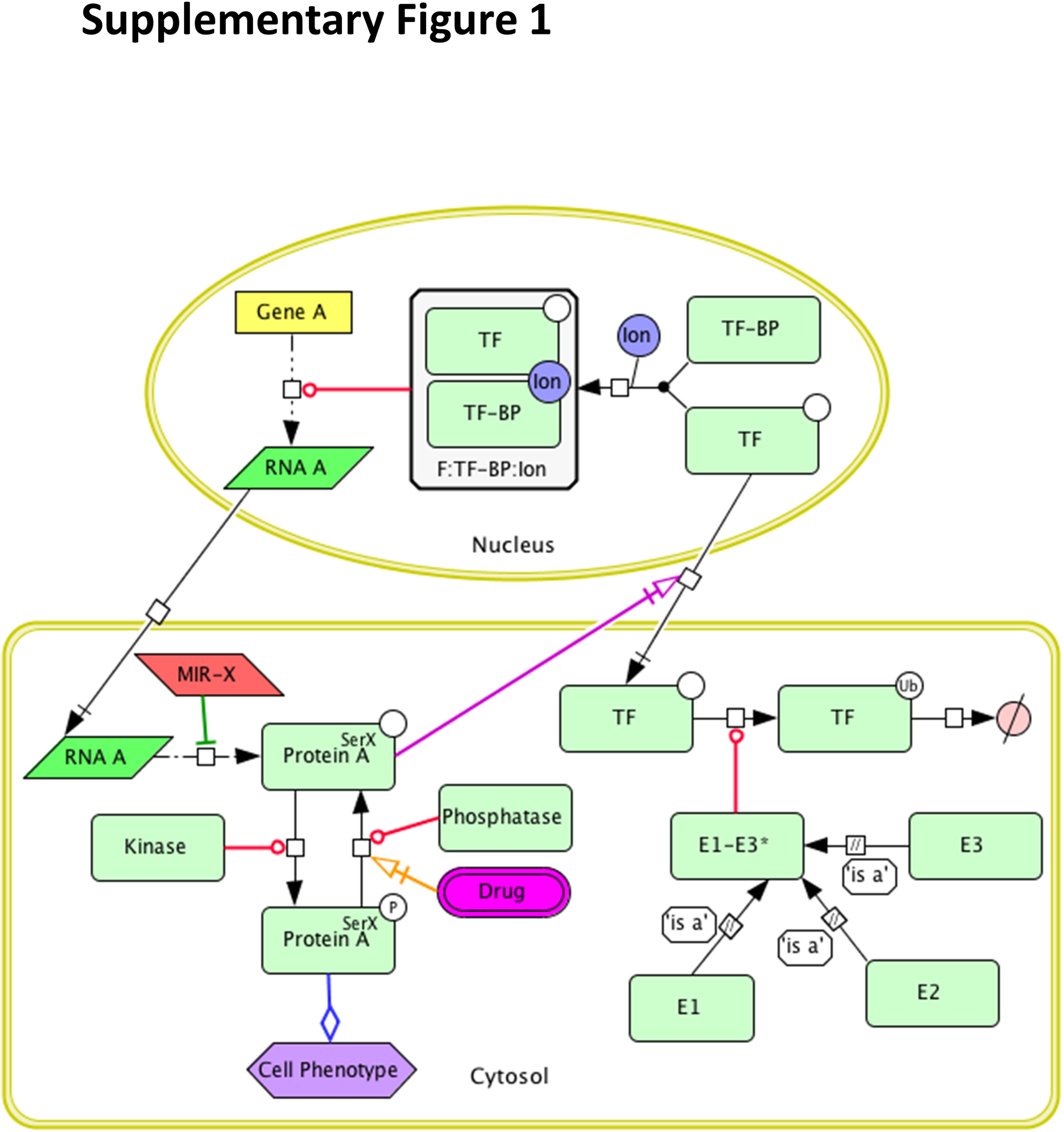

Supplement: Supplementary Figure 1 [file oncsis201519x2.tif]

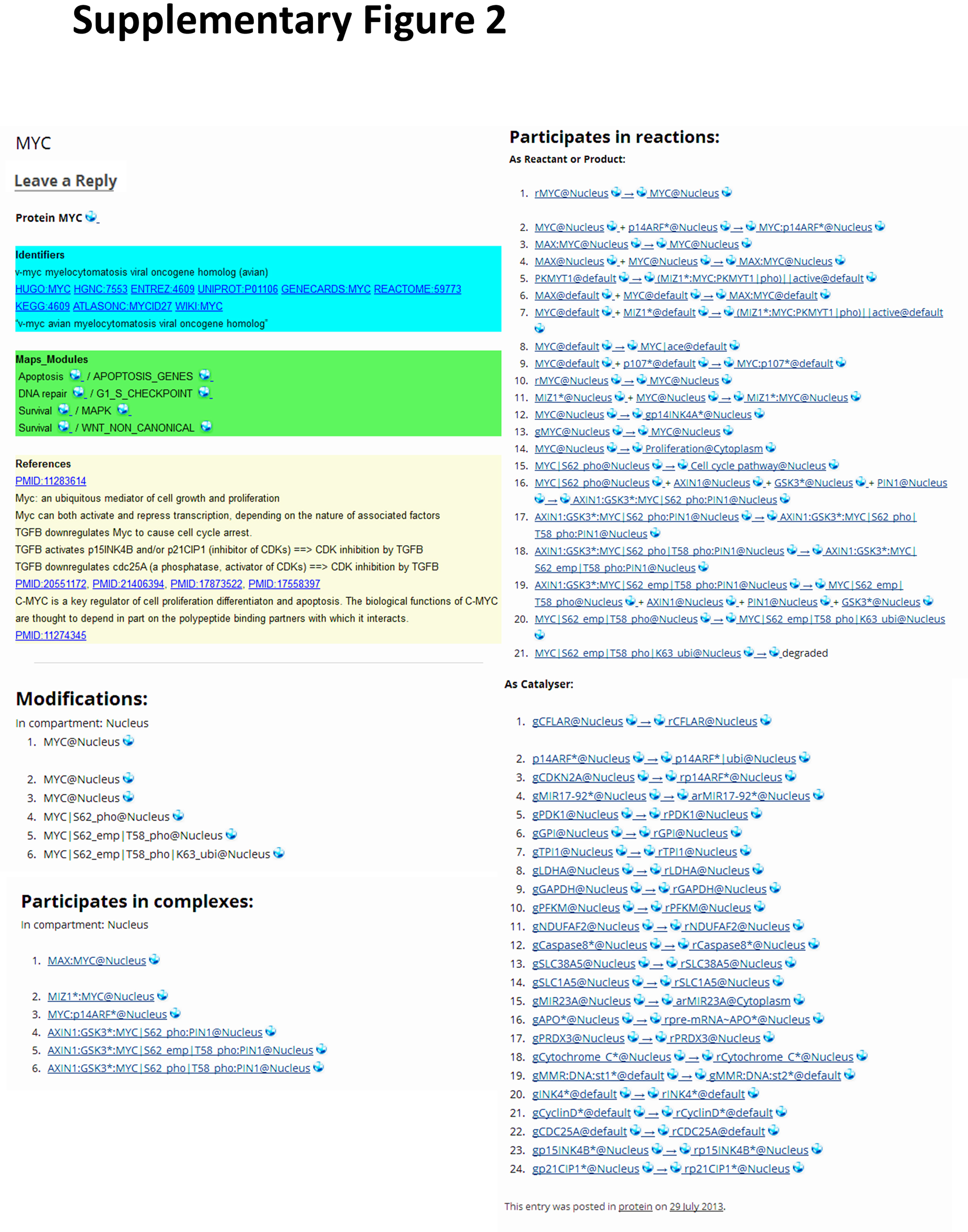

Supplement: Supplementary Figure 2 [file oncsis201519x3.tif]

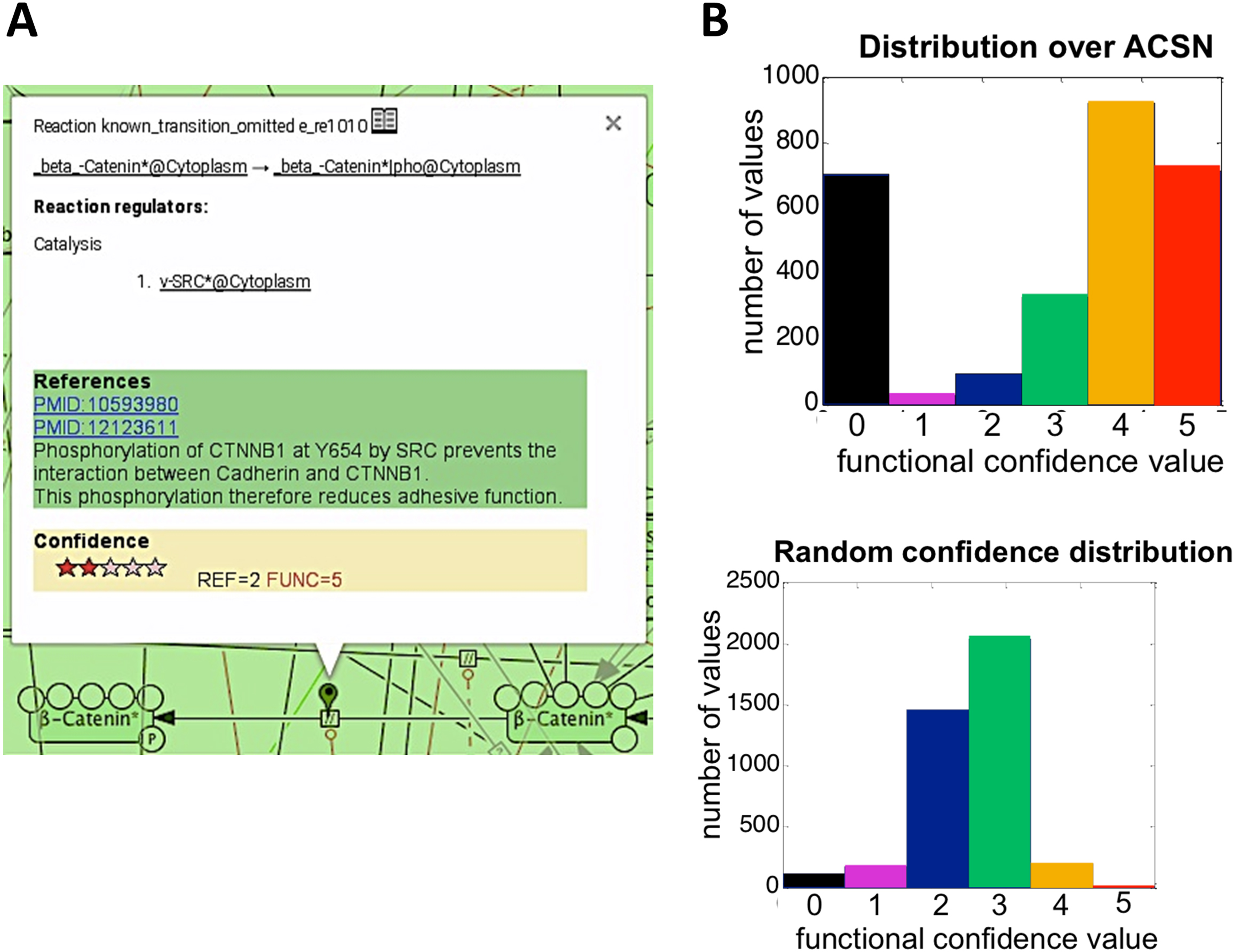

Supplement: Supplementary Figure 3 [file oncsis201519x4.tif]

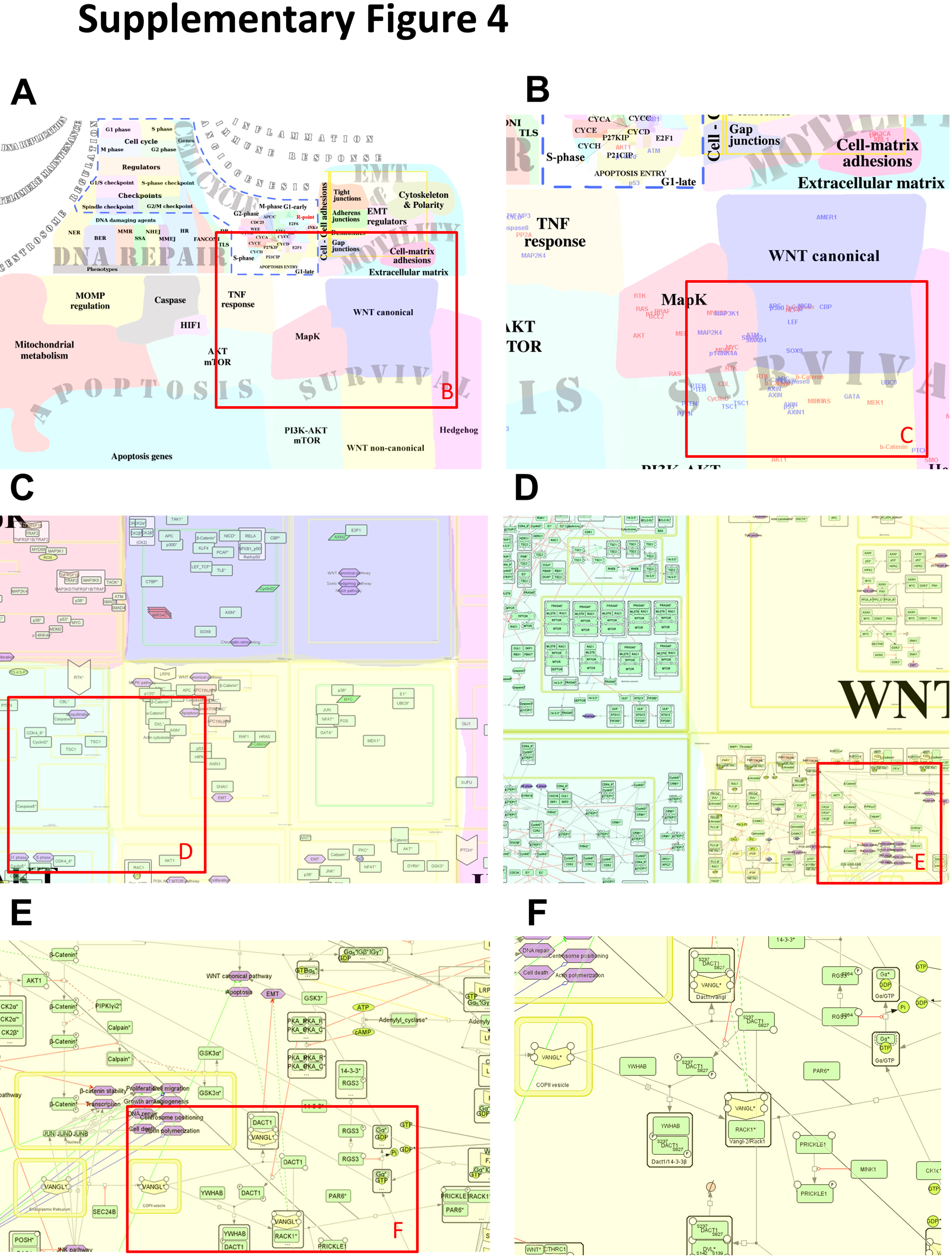

Supplement: Supplementary Figure 4 [file oncsis201519x5.tif]

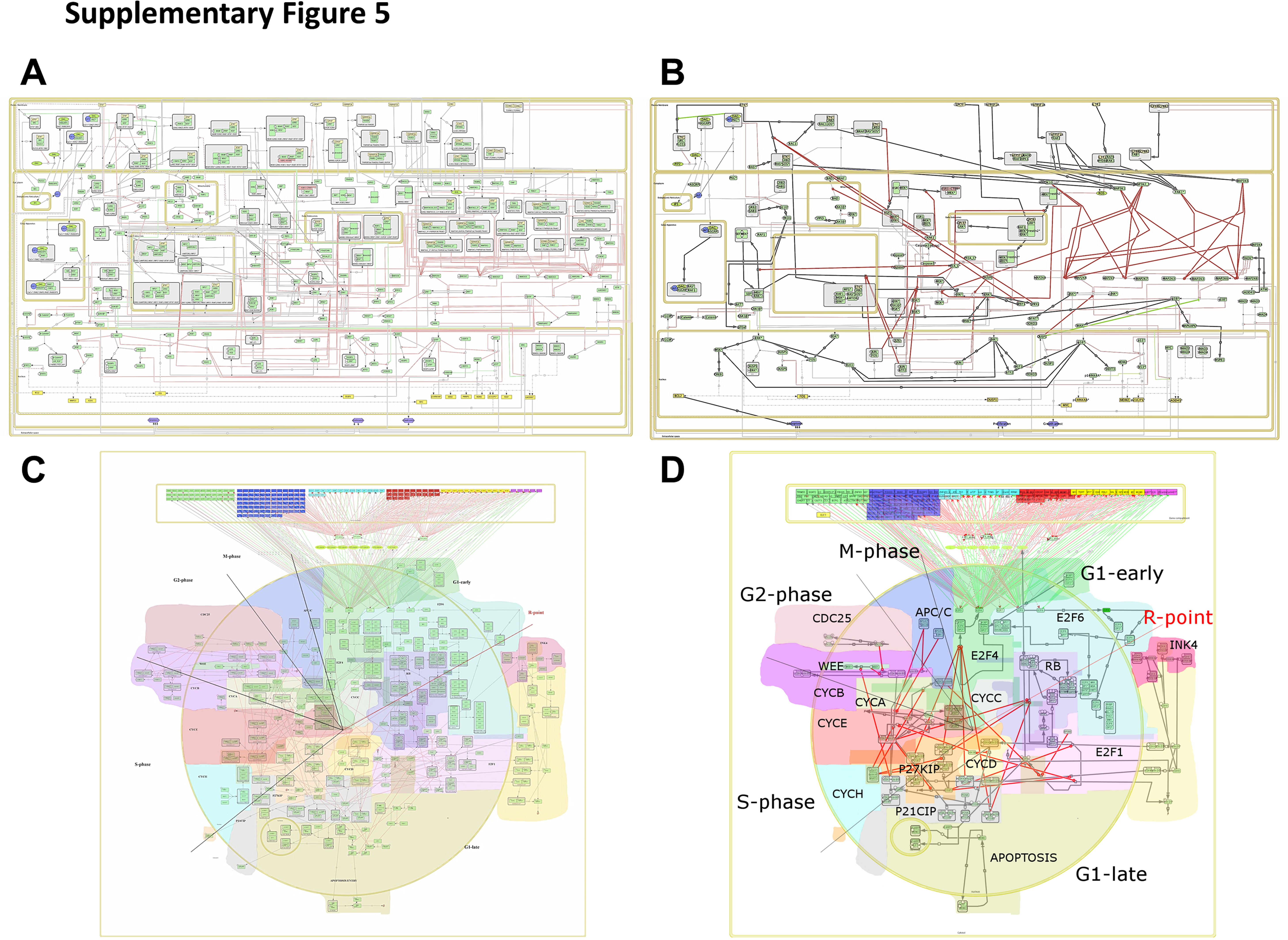

Supplement: Supplementary Figure 5 [file oncsis201519x6.tif]

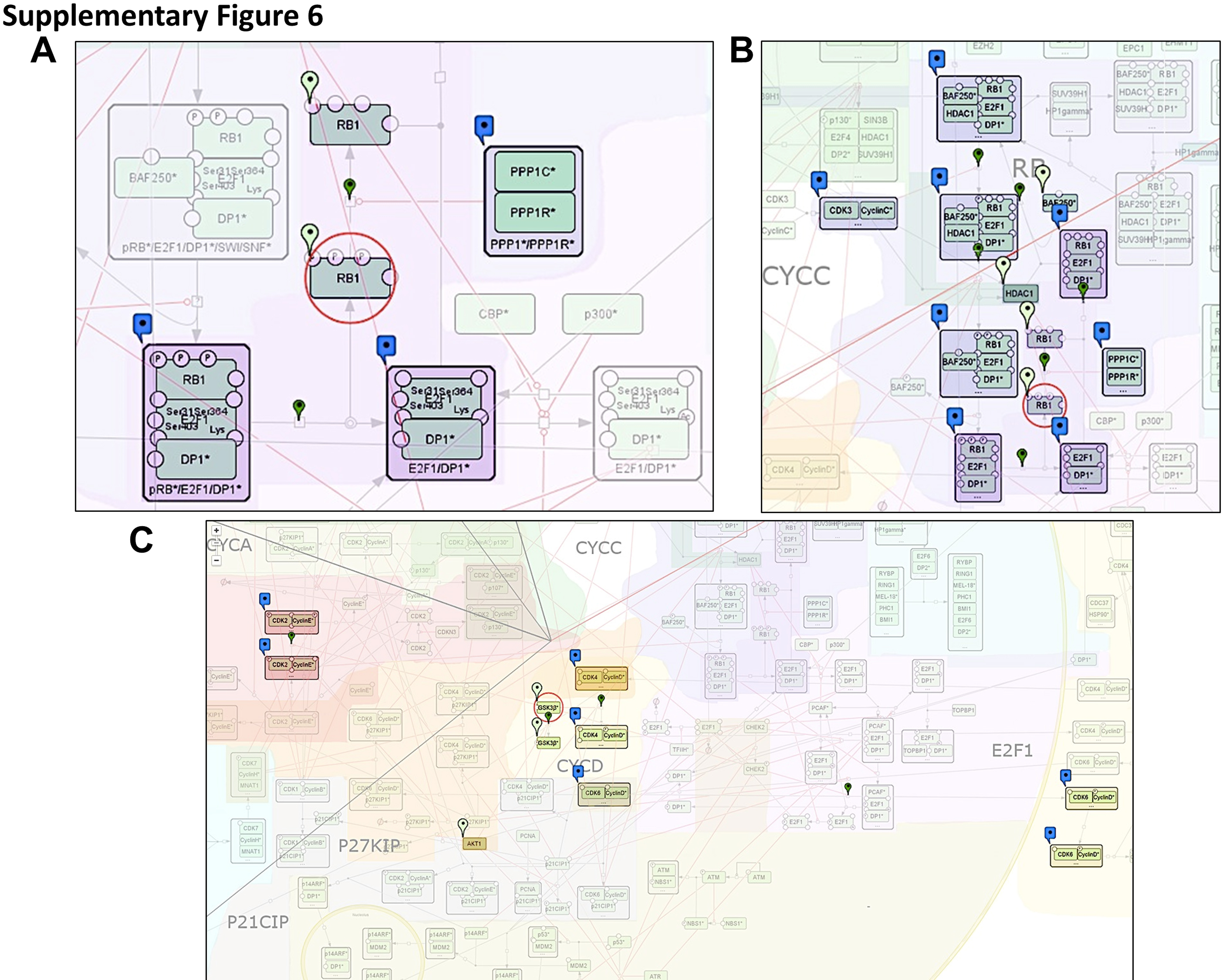

Supplement: Supplementary Figure 6 [file oncsis201519x7.tif]

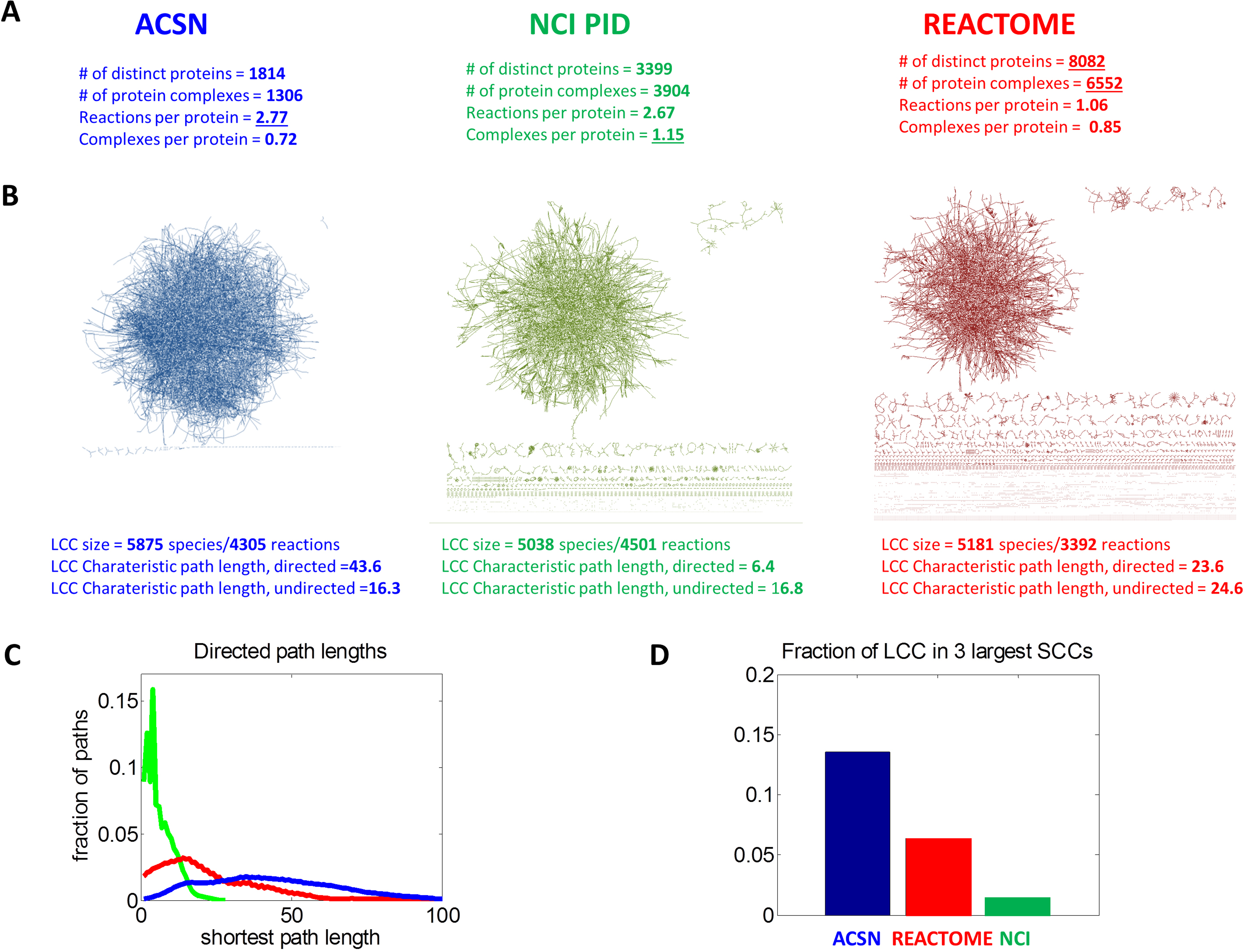

Supplement: Supplementary Figure 7 [file oncsis201519x8.tif]

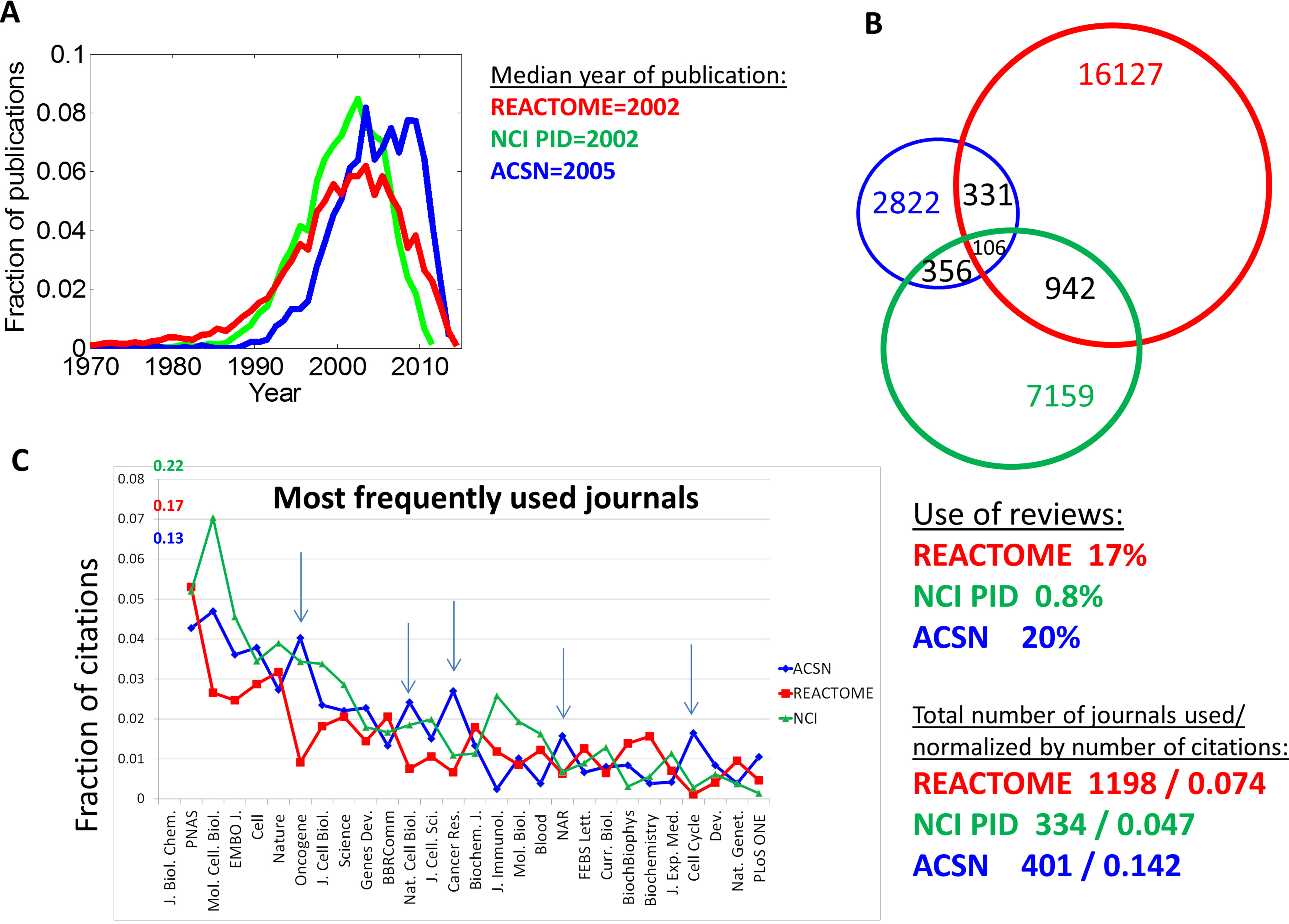

Supplement: Supplementary Figure 8 [file oncsis201519x9.tif]

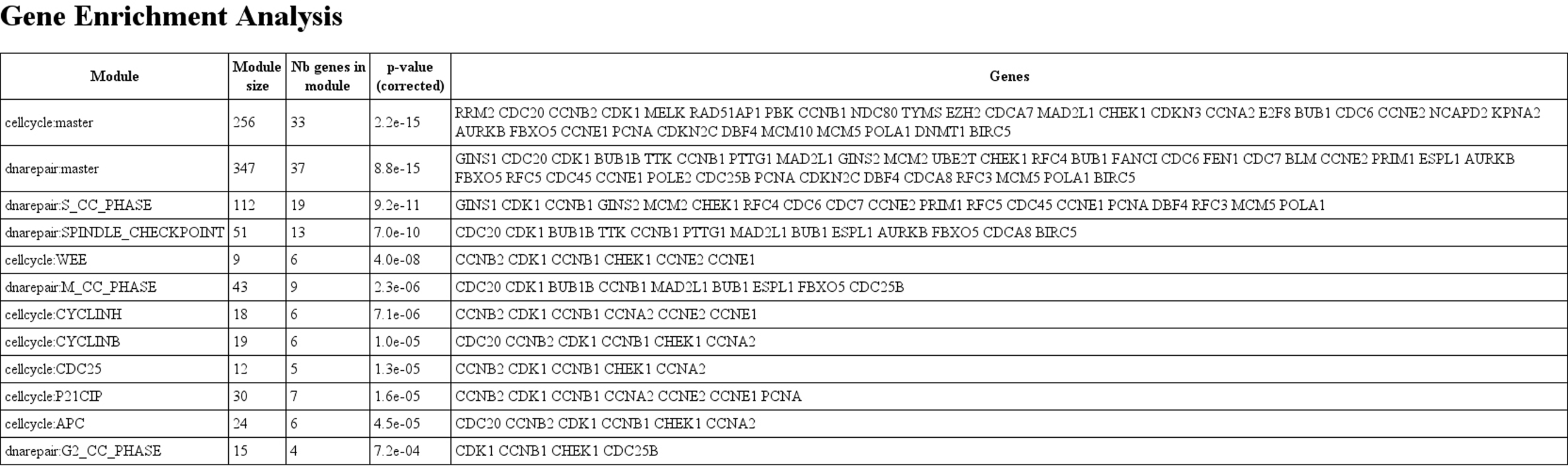

Supplement: Supplementary Figure 9 [file oncsis201519x10.tif]

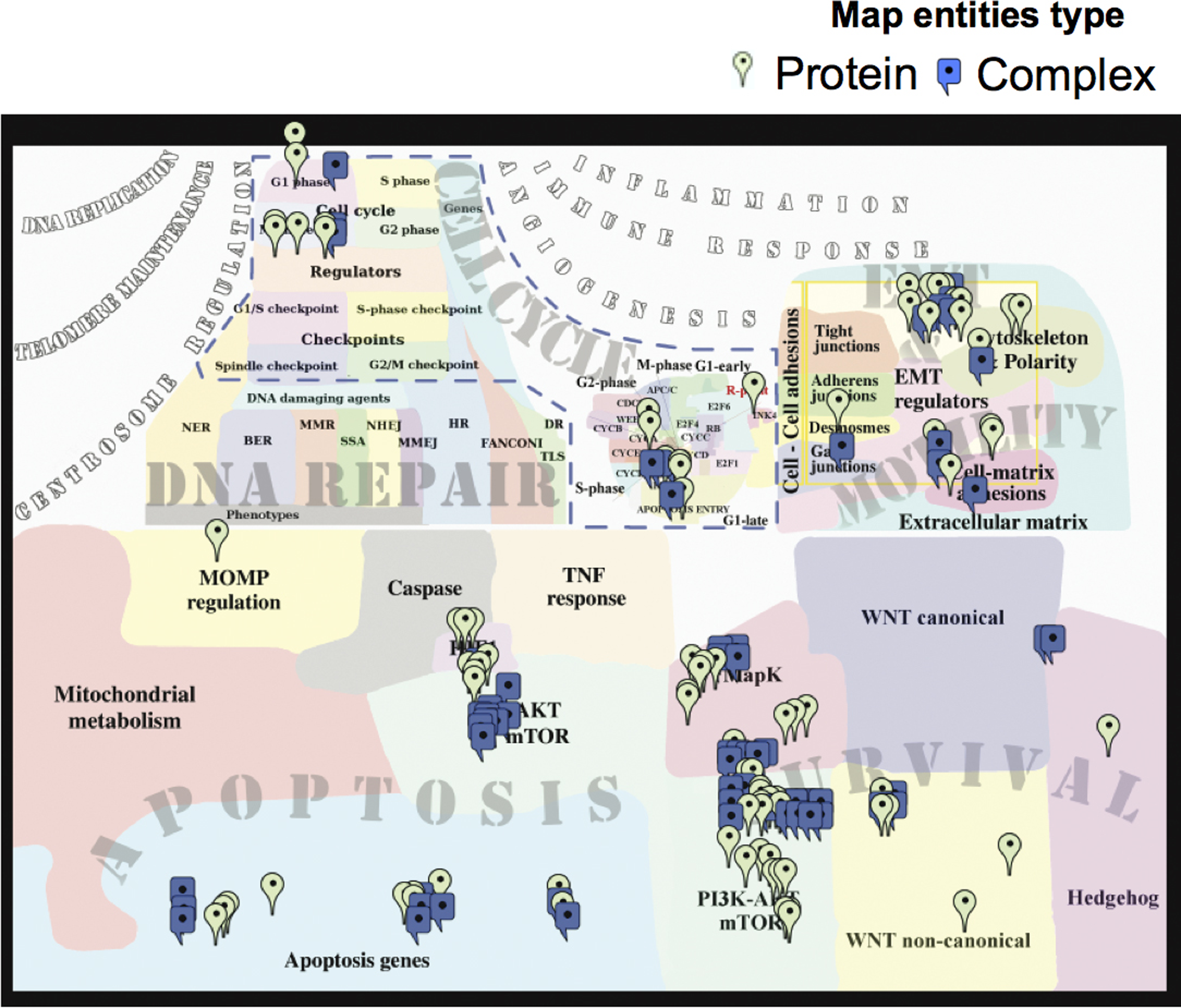

Supplement: Supplementary Figure 10 [file oncsis201519x11.tif]

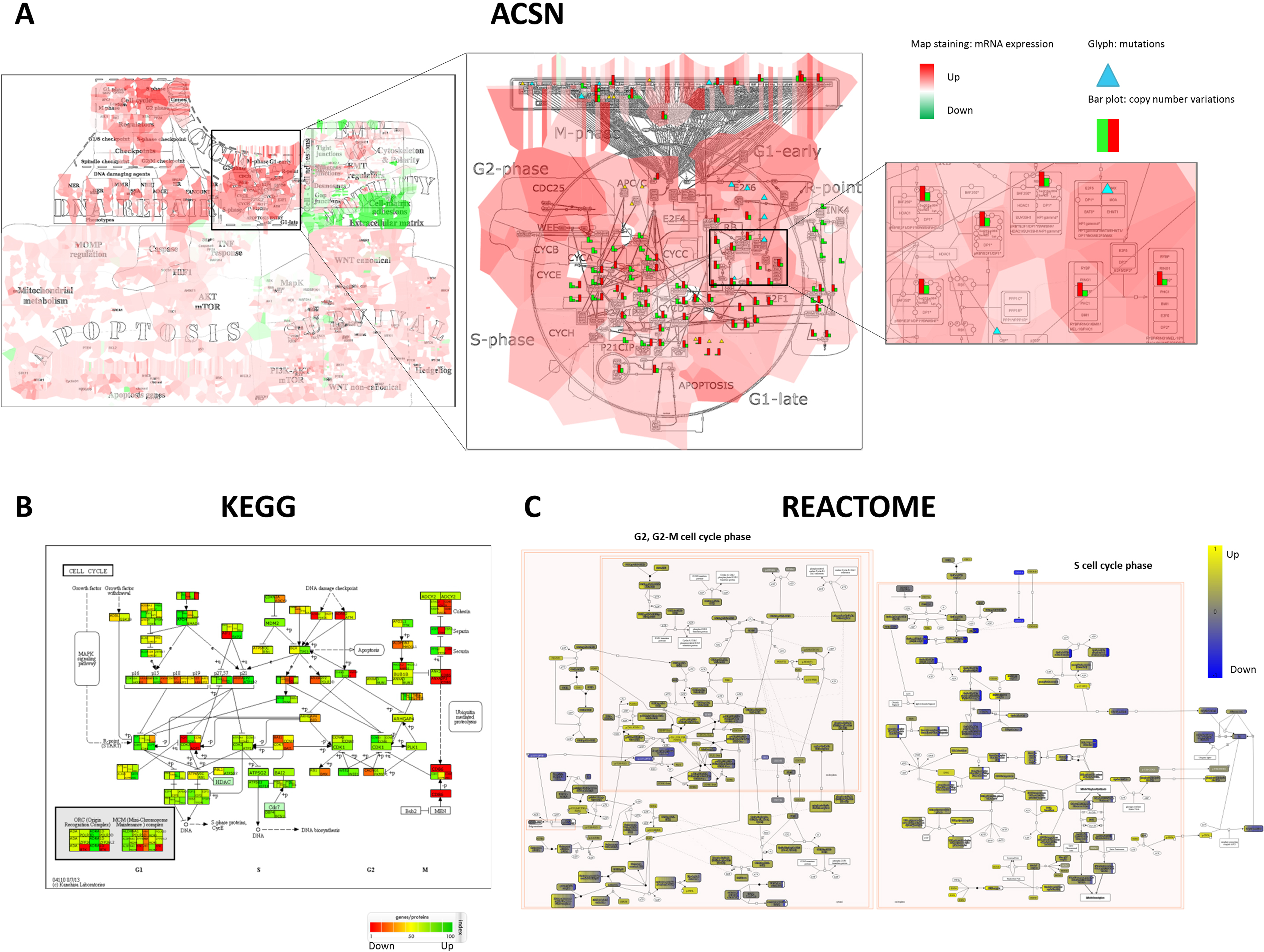

Supplement: Supplementary Figure 11 [file oncsis201519x12.tif]
